# Existing validated tools for assessing intrinsic capacity and its components within a positive ageing framework: a scoping review and structured appraisal by the Multidisciplinary International Positive Ageing Group

**DOI:** 10.1093/ageing/afag274

**Published:** 2026-09-21

**Authors:** Yves Henrotin, Luis Agüera, Giovanni Briganti, Tommy Cederholm, Demirhan Diracoglu, Gianni Franco, Alfonso J Cruz-Jentoft, Karolina Piotrowicz, Virginia Boccardi, Sandrine Vandenput, Sofia Duque

**Affiliations:** Liège University Hospital Sart Tilman Site, Department of Physical Activity and Rehabilitation Sciences, Faculty of Medicine, University of Liège, Avenue d'hippocrate 5, Liège 4000, Belgium; Instituto de Investigación Sanitaria (imas12), Hospital Universitario 12 de Octubre, Centro de Investigación Biomédica en Red de Salud Mental (CIBERSAM), Instituto de Salud Carlos III, Madrid, Spain; Universite de Mons, Department of Computational Medicine and Neuropsychiatry, Mons, Walloon Region, Belgium; Uppsala Universitet, Uppsala, Sweden; Department of Physical Medicine and Rehabilitation, Istanbul University Istanbul Faculty of Medicine, Istanbul, Turkey; Liège University, Department of Neurology, Hospital Sart Tilman Site, Liège, Belgium; Hospital Universitario Ramon y Cajal - Servicio de Geriatría, Ctra. Colmenar km 9,1, Madrid 28034, Spain; Department of Internal Medicine and Gerontology, Jagiellonian University in Kraków Medical College, Jakubowskiego 2, Kraków 31-008, Poland; University of Perugia, Department of Medicine and Surgery, P.zzale Gambuli, Perugia 06123, Italy; Health ULiège Library, University of Liège, Liège, Belgium; Internal Medicine Department, Hospital São Francisco Xavier, Centro Hospitalar de Lisboa, Lisboa, Portugal

**Keywords:** intrinsic capacity, positive ageing, healthy ageing, assessment tools, life-course approach, prevention, systematic review, older people

## Abstract

**Introduction:**

Preserving intrinsic capacity (IC) is central to healthy ageing. However, no consensus exists regarding validated multidimensional instruments suitable for assessing IC across the adult lifespan. This scoping review identified instruments used to assess IC and to evaluate their scientific validity, clinical relevance and usability.

**Methods:**

An updated literature search was conducted following the PRISMA-ScR guidelines in five databases for studies published between January 2022 and May 2025. This scoping review updates and expands upon a previously published comprehensive review. Nine experts independently evaluated all identified tools using predefined criteria: scientific validity, clinical relevance, usability in clinical practice, usability for self-assessment and suitability for adults aged <60 years.

**Results:**

A total of 61 studies describing 61 distinct IC assessment instruments were included. All five IC domains were represented, although coverage across domains was uneven. Instruments assessing sensory capacity were scarce and generally received lower ratings than those targeting other domains. Only the Generalized Anxiety Disorder-7 and EUROQOL-5D achieved the maximum expert score. Importantly, very few instruments were considered appropriate for assessing IC in adults younger than 60 years, limiting their potential use for early identification of functional decline and preventive interventions.

**Conclusions:**

Current IC assessment instruments show substantial heterogeneity in content, validity and usability, with important gaps in the assessment of sensory capacity and younger adults. The gaps identified in this scoping review suggest that the development and validation of a comprehensive, multidimensional IC assessment tool may be warranted.

## Graphical Abstract

**Graphical Abstract:**
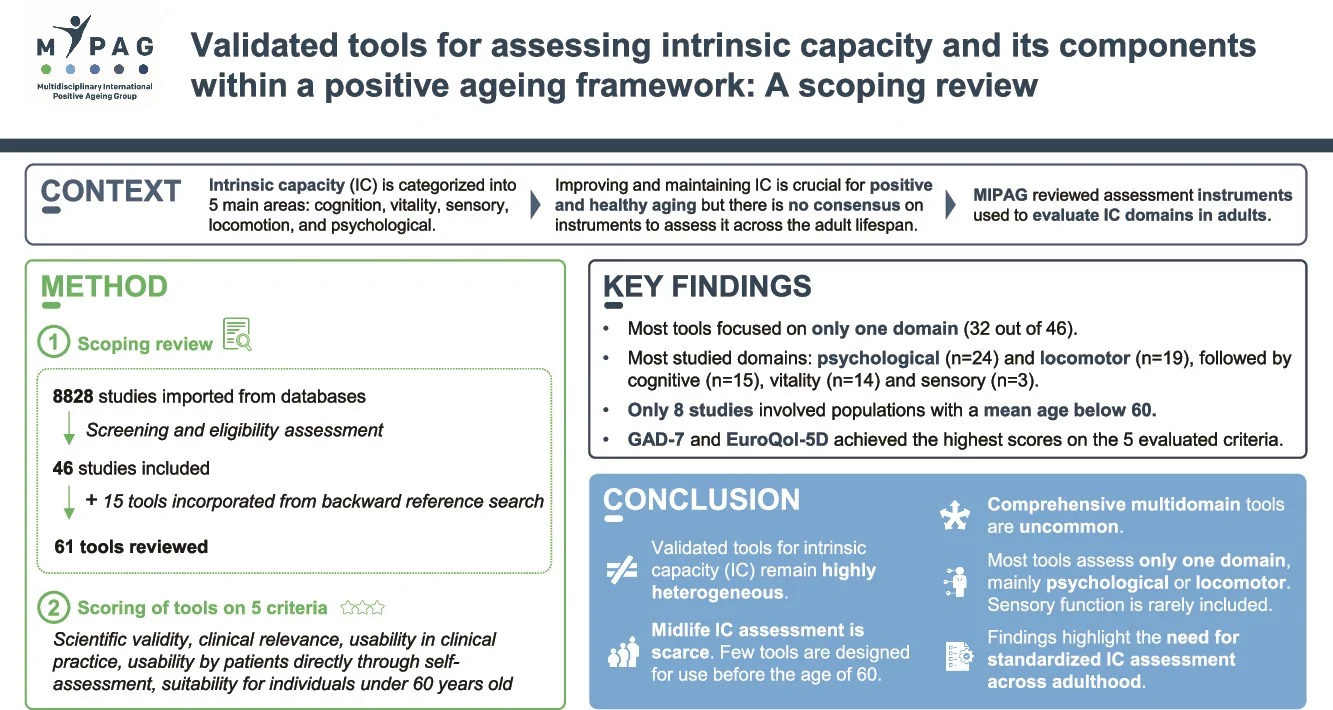

## Key points

- Intrinsic capacity assessment is a promising framework for capturing functional health beyond disease-based models, but its clinical implementation remains uneven across domains and age groups.
- Validated and feasible tools are widely available for psychological and locomotor domains
- Vitality and sensory domains remain underrepresented and insufficiently operationalised.
- Most intrinsic capacity assessment instruments are validated in older populations, highlighting a critical gap for midlife and younger adults, where early preventive interventions may be most effective.
- Expert evaluation indicates that clinical usability and applicability to individuals under 60 years are key limiting factors, even for tools with strong psychometric properties.
- Developing user-friendly, multidimensional IC tools applicable across the life course is essential to support early risk stratification, personalised interventions and proactive healthy ageing strategies.

## Introduction

In 2015, the World Health Organization (WHO) published the World Report on Ageing and Health [1], introducing a major paradigm shift in ageing science. In this report, healthy ageing was defined as the process of developing and maintaining functional ability that enables well-being in older age, adopting a holistic, functional and life-course perspective [1]. Functional ability refers to ‘the health-related attributes that enable people to be and to do what they have reason to value’ [1] and is understood as the result of the interaction between an individual’s intrinsic capacity (IC) and environmental factors. Within this framework, the WHO formally introduced the concept of IC, defined as the composite of all the physical and mental capacities that an individual can draw on [1]. IC is conceptualised as a multidimensional construct encompassing five core domains: locomotion, vitality, cognition, psychological capacity and sensory capacity. Vitality refers to the physiological energy and metabolic reserves that sustain an individual’s functioning. To operationalise this framework, the WHO introduced the Integrated Care for Older People (ICOPE) approach in 2017, which focuses on maintaining and improving IC to achieve healthy ageing [2, 3]. In this context, the WHO established in 2019 an assessment tool in the form of a mobile application, the WHO ICOPE handbook App, designed to facilitate the screening and monitoring of IC domains in clinical practice [4].

Beyond the WHO concept of healthy ageing, the broader concept of positive ageing shifts the focus from deficits and disease towards individuals’ strengths, resilience and opportunities for continued development throughout life [5]. Rather than resisting the ageing process, positive ageing emphasises adapting to age-related changes with optimism while maintaining psychological well-being, social engagement and a sense of purpose. Although early definitions primarily highlighted psychological adaptation, more recent frameworks like National Institute on Aging (NIA) have expanded the concept to encompass physical, cognitive, social and environmental dimensions across the life course. Building on this perspective, the Multidisciplinary International Positive Ageing (MIPAG) group, an international multidisciplinary expert consortium, applies structured consensus methods to develop evidence-informed frameworks and recommendations for positive ageing, with a particular focus on the life-course promotion and assessment of IC [6]. MIPAG approach aims not only to improve IC but also to strengthen resilience to physical, social, economic, psychological or emotional challenges through education, self-efficacy, self-management and health literacy. While the WHO definition of healthy ageing mainly concerns the end of life, the positive ageing framework explicitly encourages earlier and more tailored interventions [6].

One approach to promote healthy and positive ageing is the assessment of IC across its five domains, enabling early identification of functional decline and the implementation of individualised, domain-specific interventions.

To the best of our knowledge, only two reviews have summarised the available tools for assessing IC in older adults [7, 8]. The most recent review by López-Ortiz *et al*. [8] provided a comprehensive synthesis of studies up to 10 February 2022, that assessed exclusively populations over 60 years and highlighted substantial heterogeneity in IC assessment methods and the absence of consensus on global IC scoring.

According to this existing synthesis [8], locomotion and vitality were the most assessed IC dimensions, with 91% of studies evaluating them. Next were cognition and the psychological dimension, each at 88%, followed by the sensory dimension at 70%. The most frequently used tests for assessing locomotion included the Short Physical Performance Battery (SPPB) and walking speed. For vitality, handgrip strength and body mass index were the most used measurements. All studies assessing the sensory dimension relied on self-reports of hearing and visual problems, respectively. In terms of cognition, the Mini-Mental State Examination (MMSE) and the verbal fluency test were most often used. Lastly, the Geriatric Depression Scale (GDS) was the primary tool for evaluating the psychological dimension.

Therefore, an all-encompassing, integrated assessment tool covering all five domains of IC is essential for evaluating overall IC. Some studies have proposed a global or composite score that combines the various dimensions of IC. Typically, they use a composite score based on the average of scores for each dimension, an index calculated from the sum of scores across each domain, an IC score derived from a bi-factor exploratory analysis or a modified ICOPE score [9–14].

The goal of this scoping review was to examine recent studies on the assessment of IC and its domains in the general adult population, focusing on the tools used, the evaluated domains and the age groups included. Unlike previous reviews limited to adults over 60 years old, this review adopts a positive ageing and life-course perspective, emphasising that IC should be optimised early, starting in midlife. We therefore included assessment tools and scores applicable to younger adult populations and concentrated on scores and tools suitable for individual-level use in routine daily practice.

## Method

This scoping review was conducted following the Preferred Reporting Items for Systematic Reviews and Meta-Analyses extension for Scoping Reviews (PRISMA-ScR) [15]. This scoping review updates and expands upon a previously published comprehensive review by López-Ortiz *et al* [8].The final search strategy focused on concepts related to ‘healthy ageing’ AND (‘score’ OR ‘questionnaire’), as detailed in Appendix 1. The protocol was registered on the Open Science Framework (OSF: registration ID: https://doi.org/10.17605/OSF.IO/WHTZR). The full protocol has been described in the Appendix 2. Beyond descriptive mapping, the identified tools underwent a structured expert evaluation to assess their methodological robustness and practical applicability. Each tool was assessed according to five predefined criteria developed with equal weighting across criteria: scientific validity (1–5): evidence of reliability, validity and sensitivity/specificity; clinical relevance (1–5): suitability for individual-level assessment versus community/population-level assessment; usability in clinical practice (1–5): time required, complexity, training needs and feasibility in real-world settings; patient self-assessment usability (1–5): extent to which the tool can be used independently by patients without assistance from clinicians or caregivers and suitability for patients younger than 60 years (S60; 1–5): appropriateness, effectiveness and accessibility of the tool for populations under 60 years old.

The expert panel consisted of nine independent researchers and clinicians with complementary expertise in geriatrics (S.D., V.B.), neurology (G.F.), physical and rehabilitation medicine (D.D.), physiotherapy (Y.H.), psychiatry (L.A.), psychometrics (G.B.) and the assessment of intrinsic capacity (T.C., A.J.C.). To distribute the workload while ensuring independent evaluation, each assessment tool was assigned to two experts according to their area of expertise. Thus, although the panel comprised nine experts overall, each individual tool was evaluated by two independent reviewers. Before the assessment, all experts received a standardised evaluation grid describing the predefined evaluation criteria and scoring instructions to ensure consistency across reviewers. Experts completed their assessments independently using this evaluation grid and were blinded to each other’s scores during the initial evaluation. Discrepancies between reviewers were subsequently discussed until consensus was reached. When consensus could not be achieved, a third expert from the panel (Y.H.) independently reviewed the tool and contributed to the final consensus decision. Each criterion was rated on a five-point Likert scale ranging from 1 (weak) to 5 (excellent). Mean scores and standard deviations were calculated for each criterion and for the overall score, with a maximum possible total score of 25.

## Results

**Figure 1:**
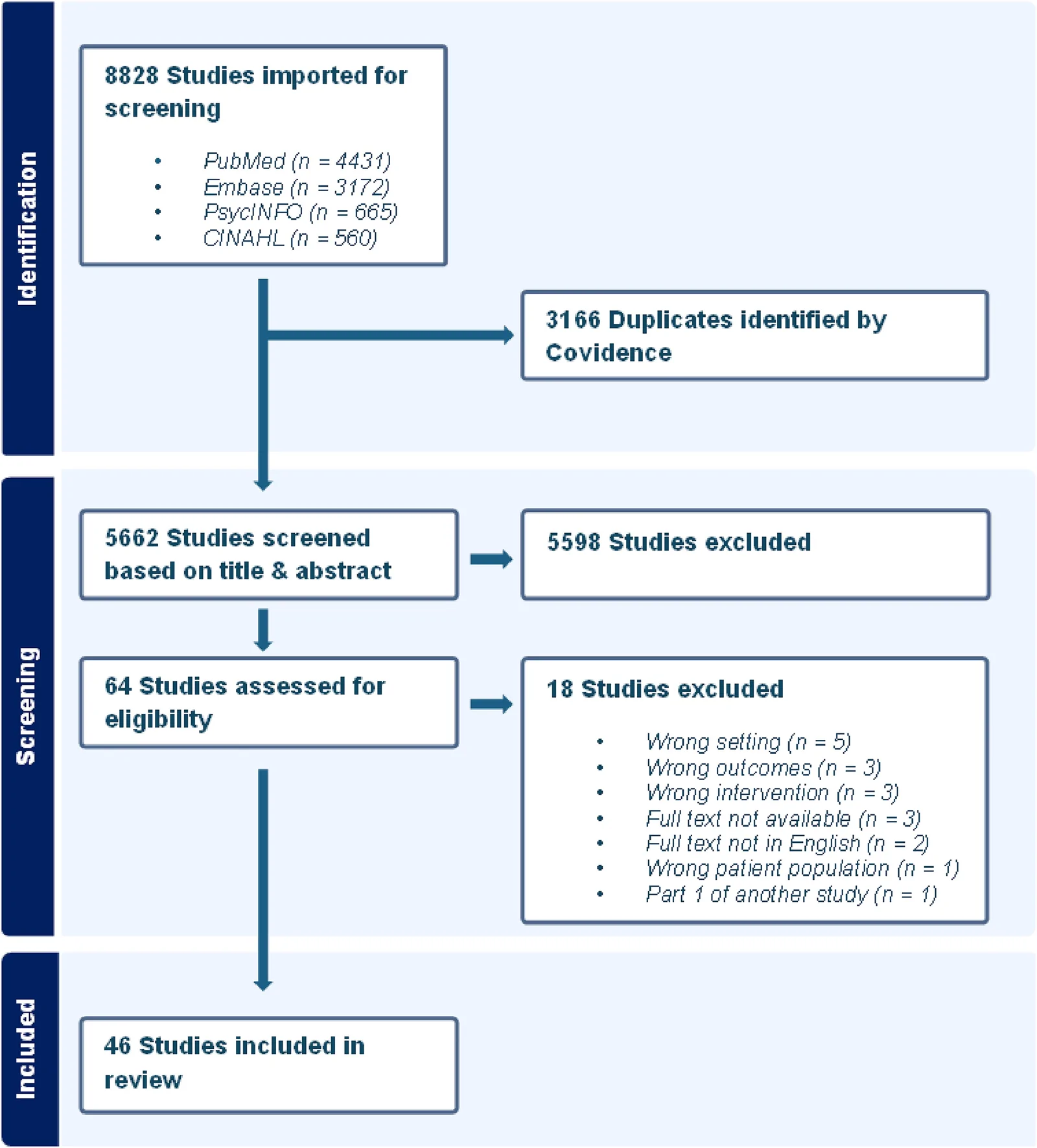
Flow diagram of the scoping review process.

The PRISMA flow diagram is presented in Figure 1.

Searches across four databases identified 8828 records. Following title/abstract and full-text screening, 46 studies describing 46 assessment tools were included (Table 1). An additional 15 studies identified by López-Ortiz *et al*. [8], covering 15 tools across the locomotor, sensory, cognitive and psychological domains, were incorporated (Table 2). Overall, 61 studies evaluating 61 assessment tools were included.

**Table 1:** Data extracted from papers of the scoping review.

First author	Year	Instruments name	Population characteristics	IC domains	Key findings
Pantuzza [16]	2023	TELUMI (Medication Literacy Test for Older Adults–Brazilian Portuguese Acronym)	30 participants. Mean age: phase A = 60.2 (7.4); phase B = 67.7 (6.3) years. Inclusion criteria: age > 60 years, self-reported ability to read and lack of cognitive, visual or hearing impairment.	Cognition	The instrument appears to have adequate content validity and is well suited to the target population. Therefore, it can help health professionals identify older adults with low medication literacy and aim to improve their medication use skills.
Ma [17]	2023	Risk-prediction nomogram (no official name)	2802 participants; mean age: 69.4 (6.8) years; 51.5% female. Inclusion criteria: age over 60 years, having completed the cognitive function test. Exclusion criteria: missing questionnaire data.	Cognition	This study provides compelling evidence of a significant association between cognitive impairment in older people and five key factors: sex, age, educational level, engagement in moderate-intensity physical activity in daily life and the presence of taste problems.
Oxbøll [18]	2024	Brief Assessment of Impaired Cognition– Questionnaire (BASIC-Q)	275 participants; mean age: 77.6 (5.4) years; 53% female. Inclusion criteria: ≥70 years, had a close relative or friend who agreed to serve as an informant about the participant’s activities of daily living, sufficiently fluent in Danish to participate in cognitive testing without the assistance of an interpreter. Exclusion criteria: known diagnosis of dementia, or a condition that, in the judgement of the GP, would affect the subject’s ability to cooperate in the extended cognitive assessment, such as addiction to drugs, alcohol or medication; severe chronic psychiatric or physical illness; or significant sensory deficits. Overall, the expert appraisal identified only a limited number of instruments that combined strong psychometric properties with high clinical usability. The GAD-7 and EUROQOL-5D achieved the highest overall score (25/25), reflecting excellent scientific validity, clinical relevance and ease of implementation. In cognition, the highest-rated tools were MMQ-9, the Risk Prediction Nomogram and BASIC. In locomotion and vitality, only the BPAAT and IPAB, respectively, scored above 20/25. Within the psychological domain, the highest-rated tools were the GAD-7 (25/25), F-SOZU K-6 (24/25) and the Geriatric Depression Scale (22.5/25). Sensory tools received lower ratings overall, with the HHIE-S outperforming the Frisby Stereo Test (18.5 vs. 10.5/25). Among multidomain instruments, the EUROQOL-5D, CESAM Questionnaire and Comprehensive Frailty Assessment Instrument (CFAI) obtained the highest overall scores. Self-administered questionnaires generally achieved higher usability scores than performance-based assessments or prediction models. Importantly, despite the inclusion of 61 assessment instruments, only a small proportion were considered suitable for adults younger than 60 years, reinforcing the current lack of tools capable of detecting early declines in IC before older age. Sensory and vitality domains also remained underrepresented, highlighting important gaps for future instrument development.	Cognition	BASIC-Q is a brief, easy-to-use questionnaire for the identification of cognitive impairment in older adults. It demonstrated fair-to-good classification accuracy in a general practice setting and can be a useful case-finding tool when suspecting dementia in primary health care.
Jørgensen [19]	2024	Brief Assessment of Impaired Cognition (BASIC)	255 participants; normal cognition patients (*n* = 154; mean age: 76.5 (5.2) years; 57% female; cognitive impairment patients (*n* = 101; mean age: 79.3 (5.1) years; 48% female. Inclusion criteria: patients from GP clinics aged 70 years and over; being fluent in Danish; having a close relative agreeing to complete the informant report in BASIC. Exclusion criteria: a known diagnosis of dementia or a condition that, judged by the physician, would affect the patient’s ability to complete the comprehensive cognitive assessment.	Cognition	The BASIC Danish version demonstrated good discriminative validity in a general practice setting. Classification accuracy of BASIC is equivalent to more complex, time-consuming instruments (MMSE, MOCA and RUDAS) and higher than very brief instruments (CDT-6, MINI-COG and BASIC-Q).
Troyer [20]	2025	Multifactorial Memory Questionnaire 9 items (MMQ-9)	Study 1: 560 participants; mean age: 71.3 years; 69% women; Study 2: 638 participants; mean age: 73.2 years; sex ratio: 71% women. Inclusion criteria: study 1: scored within normal range on a cognitive screening test; study 2: age > 50; ability to complete the study questionnaires in English.	Cognition	The shortened MMQ-9 is a reliable, valid and invariant measure of metamemory in middle-aged and older adults.
O’Donovan [21]	2023	Cognitive Screening Instrument for the Survey of Health, Ageing and Retirement in Europe (SHARE-COG)	20,752 participants; mean age: 74.7 (6.6) years; 55% female Inclusion criteria: aged ≥65 years who completed a longitudinal questionnaire in wave 8 (2019/20) of the SHARE. Exclusion criteria: participants missing data for the variables of interest, physically unable to complete the cognitive drawing tests, or who could not be reliably classified into one of the cognitive diagnostic groups.	Cognition	In conclusion, SHARE-Cog performed well, demonstrating good-to-excellent diagnostic accuracy across the spectrum of cognitive impairment in this validation study.
Al-Heizan [22]	2022	Menu Task	287 participants; mean age: 69.7 (8.8); 73.6% women. Inclusion criteria: community-dwelling adults aged 55 years or older, with both verbal and written understanding of the English language. Exclusion criteria: living in skilled nursing or assisted living facilities, or lacking the vision, hearing or motor skills required for testing.	Cognition	Both concurrent and construct validity were supported. The Menu Task demonstrates sensitivity to functional cognitive impairments in a community sample.
Guevarra [23]	2025	Hand Grip Strength (HGS) measured with an aneroid sphygmomanometer	70 participants; mean age: 65.7 (9.6); 74.3% female. Inclusion criteria: 50 years old and above, with no hand orthopaedic problems or disability. Exclusion criteria: psychiatric, psychological and/or mental disabilities and unstable comorbidities that the HGS may aggravate.	Locomotor	An aneroid sphygmomanometer can be used to measure hand grip strength and has a valid predictive value for the Jamar hand grip strength. Hence, it can serve as an alternative tool for diagnosing sarcopenia.
Yang [24]	2025	electronic Short Physical Performance Battery (eSPPB)	58 participants; mean age: 75 (5.5); 81% women; mean body mass index (BMI): 24.2 (3.8) kg/m2. Inclusion criteria: age: 65 and older, able to ambulate at least 100 m independently, with an Abbreviated Mental Test (AMT) score of at least 6, and who could understand instructions and adhere to the study protocol Exclusion criteria: participants with a diagnosis of Parkinson’s disease, life-limiting illness (such as cancer or end-organ heart, lung, liver or renal failure or dementia)	Locomotor	The study corroborates construct validity, inter-rater agreement and test–retest reliability between the eSPPB kiosk and mSPPB in healthy community-dwelling older adults.
Li [25]	2025	Brief Physical Activity Assessment Tool (BPAAT)	—Phase 1: 75 participants; mean age 32.7 (9.8); 75% female. —Phase 2: 106 participants; mean age 37.1 (11.6); 87% female. —Phase 3: 53 393 participants; mean age 37.2 (14.2), 45% female. Inclusion criteria: —Phase 1: adults with limited resources; from a household having at least 1 child aged <19 years; aged ≥18 years; have sufficient English proficiency to read, understand and respond to the questionnaire without assistance; and have no previous participation in Expanded Food and Nutrition Program (EFNEP). —Phase 2: EFNEP participants who spoke English and who were able to walk without assistance. —Phase 3: all participants who completed both pretest and posttest BPAAT assessments.	Locomotor	The test–retest reliability of the Brief Physical Activity Assessment Tool was moderate. Concurrent validity varied across questions, with the highest value for the muscle-strengthening question. All questions demonstrated moderate sensitivity to change and responsiveness. Patterns between non-Hispanics and Hispanics were similar. The study resulted in a brief, validated, nationally tested questionnaire to evaluate changes in physical activity among non-Hispanic adults with limited resources, suitable for inclusion in short surveys. It could be further validated among other populations.
Zhou [26]	2024	Novel score for predicting 1-year falls	334 participants; mean age: 69.4 (6.25) years; 65.3% female. Inclusion criteria: aged >60 years and attending the fall clinic from January 2019 to January 2021. Exclusion criteria: aged <60 years; incomplete questionnaire and evaluation data or missing relevant information records; and stroke, Alzheimer’s disease or other medical diseases that affected activity.	Locomotor	The newly developed score demonstrated greater accuracy in predicting falls among older adults than the Timed Up and Go test and the 30-Second Chair Sit-Stand test. Additionally, the scale showed superior clinical validity for identifying fall risk.
Warabino [27]	2024	The Self-Assessment Burden Scale–Motor (SAB-M)	168 participants; mean age: 78.1 (11.5) years; 44% male. Inclusion criteria: living at home for at least 3 months because the recovery rate has been reported to be more stable starting from 3 months after discharge, being cared by healthy family caregivers and receiving home rehabilitation services. Exclusion criteria: being cared for by family caregivers with dependent ADL and dementia.	Locomotor	The SAB-M has sufficient reliability and validity among community-dwelling older adults. Family caregivers can routinely assess changes in the ADL of community-dwelling older adults using the SAB-M, enabling them to consider introducing rehabilitation when older adults’ ADL declines promptly. Therefore, implementing SAB-M helps older adults live and function at home for as long as possible.
Poncumhak [28]	2022	Circular tandem walk test (CTWT)	89 participants; mean age: 74.2 (5.6) years; 51.7% female Inclusion criteria: aged 65 years and older, able to walk without an assistive walking device at least 10 steps, understand the commands used in this study and perform activities of daily living independently. Exclusion criteria: neurological signs or any serious diseases that might affect balance, significant pain in the lower extremities or other parts of the body that might affect participation (scoring more than five on the Visual Analog Scale), uncorrected visual deficits or amputation of a lower extremity.	Locomotor	The Circular Tandem Walking Test (CTWT) is better at identifying risk and fear of falling in older people living at home than the straight-line conventional Tandem Walking Test (CTWT). A CTWT ≥ at 14.6 s is the optimal cutoff for determining fall risk in community-dwelling older adults.
Jabbar [29]	2023	Algorithm for Measurement of Sedentary Behaviour	20 participants; mean age: 81.1 (6.2) years; > 60% women. Inclusion criteria: age of 75 years or over, able to ambulate a minimum of 15 m independently, with or without walking aids, able to stand, with or without walking aids, for a minimum of 60 seconds. Exclusion criteria: any significant medical, orthopaedic or neurological conditions that would contraindicate normal activity, allergy to surgical adhesive tape	Locomotor	These results indicate good to excellent agreement for the novel algorithm, providing a valid measure of sedentary behaviour in community-dwelling older adults.
Wingood [30]	2022	Social Ecological Model-based Inventory of Physical Activity Barriers Scale (IPAB)	503 participants; mean age: 70.0 (8.5); 69.8% female. Inclusion criteria: Community-dwelling adults 50 years and older, able to read, comprehend and write in English. Exclusion criteria: individuals who needed assistance from another person to leave their house or lived at an assisted living or long-term care facility.	Vitality	The previously published IPAB questionnaire has been refined and validated: the 27-item refined scale is internally consistent, has high test–retest reliability and can differentiate between individuals who met the recommended levels of physical activity and those who did not.
Binh Duong Thai [31]	2023	Telephone administration of the Simplified Nutritional Appetite Questionnaire (T-SNAQ)	120 participants; mean age: 78.0 (5.8) years; 59.2% female. Inclusion criteria: community-dwelling older adults aged ≥70 years, with availability of a scale to measure body weight. Exclusion criteria: Any visual and auditory impairments.	Vitality	The T-SNAQ is a feasible screening instrument for anorexia of ageing in community-dwelling older adults via telephone interviews.
Fulay [32]	2024	Nutrition Equity Index (NEI)	2468 participants; mean age: 74.7 (2.9) years; 52% female. Inclusion criteria: participants of the health, ageing and body composition longitudinal study (aged 70–79) with no difficulty walking ¼ mile, climbing 10 steps or performing activities of daily living, had no cancer or active treatment in <3 years and planned to remain in the study area for at least 3 years.	Vitality	NEI performed similarly to the previously validated United States Department of Agriculture (USDA) reference measure of food insufficiency. Food insufficiency was associated with lower dietary quality after multivariable adjustment.
Foo [33]	2024	AUTOMAL (automated malnutrition screening model)	539 participants; mean age: 84.5 (8.5) years; 62% of women. Inclusion criteria: residents in long-term care facilities. Exclusion criteria: residents in memory support units or designated dementia units, unwilling to participate, not present at the facility or unavailable during the period of data collection (July–Sept 2022 and March–Sept 2023)	Vitality	The AUTOMAL demonstrates excellent ability to differentiate malnutrition status. It makes automated identification of malnutrition possible by using two variables commonly found in electronic health records (BMI and weight change % over 6 months)
Steinman [34]	2022	Novel multimorbidity index (name not reported)	5102 participants; median age: 76 (71–80), 58.7% women. Inclusion criteria: age > 67, adults who participated in the 2010 United States Health and Retirement Study (HRS).	Vitality	A new series of claims-based multimorbidity measures was modestly better at predicting hospitalisation and functional decline than several legacy indices and no better at predicting death. There may be limited opportunities in claims data to measure multimorbidity better than older methods.
Wilson [35]	2022	Resilience Scale for Older Adults (RSOA)	Study 1: 345 participants; mean age = 65.32 (4.6) years; 66.2% female; residing in Canada and the USA and from Amazon’s Mechanical Turk. Study 2: 216 participants; mean age 71.5 (7.8) years; 65.7% female; community-dwelling adults living in Canada. Study 3: 365 participants; 69.3% female; mean age: 64 (5.3) years. Inclusion criteria: study 3: age > 55 years old.	Psychological	The RSOA is a promising new multidimensional measure that may offer a range of theoretical and applied uses for examining protective factors of resilience in an older adult population.
Noordink [36]	2024	Psychological Empowerment Scale for Older People (PESOP)	398 participants; mean age: 74.8 (15.2) years; 48% women; education level: 2% had completed no or only primary education, 25% had secondary education as their highest level of education and 73% had completed college or university education. Inclusion criteria: 65 years or older	Psychological	This study provides a valid and reliable measure for evaluating the extent to which older people perceive themselves as empowered. The relatively small number of items in the scale and its unidimensionality suggest that it is easy to administer and understand, qualities that may be particularly valuable if there is a desire to avoid unnecessarily burdening the target population.
Stewart [37]	2025	Age Causes Illness Scale	347 participants; 60% female; mean age: 55.9 (8.7) Inclusion criteria: registered voter, aged 45–65, urban-dwelling	Psychological	The seven-item Age Causes Illness scale is reliable and shows expected discriminant and convergent correlations with relevant socio-demographic, psychosocial and ageing-related measures. The belief that ‘age causes illness’, as assessed by this new scale, is associated with both objective and subjective indicators of physical health. The Age Causes Illness scale is a brief screening tool that may be applicable in behavioural health settings as an initial step towards discussing the implicit, and often unchallenged, belief that age alone determines the onset, progression and offset of illness.
O’Shea [38]	2025	Depression, Anxiety, and Apathy (DA3) scale	252 participants; mean age: 68.7(9.4) years; 65.5% female Inclusion criteria: Minimum of 45 years of age, diagnosis of no cognitive impairment, subjective cognitive impairment, mild cognitive impairment and any cause of dementia at the mild stage, defined as a Clinical Dementia Rating (CDR) of 0.5 or 1, ability to identify a study partner, be eligible to undergo an MRI exam and have complete data for all mood measures. The study is designed to include older adults, but allowances are made for midlife adults with early-onset disease, a strong family history or other dementia risk factors. Exclusion criteria: did not consent to data or biospecimen storage, not diagnosed with moderate or severe dementia (CDR 2 or 3), or had a significant medical disorder or illness that could affect longitudinal participation, or obfuscate cognitive or imaging outcomes (e.g. cancer, unstable diabetes or hypertension, substance abuse) or axis I psychiatric disorders (e.g. major depressive disorder, bipolar disorder) developing before age 50.	Psychological	The DA3 is a unitary, brief, valid and reliable tool for assessing depression, anxiety and apathy in community-dwelling older adults, including those with cognitive impairment.
Davies [39]	2024	The Emoji Current Mood and Experience Scale (ECMES)	Study 1: 672 participants; mean age: 33.3 (6.4) years; 52.4% female. Study 2: 415 participants (among whom 212 provided data at a second time point); mean age: 24.3 (±3.2), 71% male Inclusion criteria: Study 1: >18 years of age, speaking English as their first language; Study 2: individuals attending a structured 6- or 8-week (1 day per week) sustainable construction project for ‘disadvantaged’ and ‘hard to reach’ people during the period January 2018 to December 2019 and >16 years of age	Psychological	Multidimensional scaling revealed relationships with existing scales, demonstrated a meaningful structure in the emoji measure and its validity. Stability over time and sensitivity to change were also shown.
Jarsch [40]	2022	Social Inference Test–Emotion Recognition (BASIT-ER)	240 participants; aged between 35 and 92 years; 50% women. Inclusion criteria: cognitively and mentally healthy, Central European participants with age ≥ 35 years; a minimum total education of 7 years; German and/or Swiss, German as mother tongue; self-report of good health Exclusion criteria: signs of depressive mood; cognitive deficits; systemic or brain disease; psychiatric disorders; traumatic brain injury; chronic pain; history or current regular intake of any psychoactive drugs (except benzodiazepines for sleep); general anaesthesia within the last 3 months; severe sensory or motor deficits.	Psychological	The first German-language adaptation of The Awareness of Social Inference Test–Emotion Evaluation Test (TASIT-EET), called the BASIT-ER, was preliminarily validated in healthy adults.
Nam [41]	2024	SMILE (Senior Meaning in Life Evaluation)	2038 participants; mean age: 77.9 (5.6); 46.7% female; 20.0% disease; no disease; 46.7% chronic disease; 33.3% nervous system disease. Inclusion criteria: 3 samples: 1: age 60 and + using senior welfare centers in Seoul; 2: age 60+, senior welfare centers in Seoul; 3: senior citizens, from Korea Provinces	Psychological	Using the four factors and 18 items extracted by exploratory factor analysis, convergent validity was assessed, and the SMILE was significantly and positively correlated with the Rosenberg Self-Esteem Scale and the World Health Organization Quality of Life–Brief version (WHOQOL-BREF), and showed a significant negative correlation with the Center for Epidemiological Studies-Depression Scale (11-item). Concurrent validity results also showed a high positive correlation with the Meaning in Life Questionnaire (MLQ).
Smith [42]	2024	Upstream Social Interaction Risk Scale (U-SIRS-13)	4082 participants, mean age: 69.6 years; 58.5% female Inclusion criteria: adults aged 60 years and over recruited in the USA via a Qualtrics internet panel	Psychological	The U-SIRS-13 is a multidimensional scale with strong face, content and construct validity. Findings support its practical utility to identify threats to social connectedness among older adults posed by limited physical opportunities for social interactions and a lack of emotional fulfilment from social interactions.
Shah [43]	2023	Social Frailty Index	8250 participants (4302 in the 2010 development cohort and 3948 in the 2012 validation cohort); median age: 75 [70, 80] years in the 2010 cohort and 75 [71, 80], 57% female in the 2010 cohort, 58% in the 2012 cohort. Inclusion criteria: adults aged ≥65 years from the Health and Retirement Study who completed the Psychosocial and Lifestyle Questionnaire in 2010 or 2012.	Psychological	The Social Frailty Index accurately risk-stratifies older adults and improves the prediction of commonly used comorbidity- and function-based risk models.
Reinwarth [44]	2024	Perceived Social Support Questionnaire (F-SOZU K-6)—Brief form (6 items)	706 participants; mean age: 71.1 (7.2); 49.3% female. Inclusion criteria: over 60 years old. Exclusion criteria: missing data for the FSOZU K-6.	Psychological	The F-SOZU K-6 is a reliable and economical tool to assess perceived social support in older adults. Norm values for individuals over 60 years are provided
Husain [45]	2025	Gerascophobia or Excessive Fear of Ageing Scale (GEFAS)	1594 participants; mean age: 34 years; 44% female Inclusion criteria: Participants from Pakistan aged 18 and over	Psychological	The GEFAS demonstrated high reliability and validity. Significant positive correlations were found between fear of ageing and depression, anxiety, stress, death anxiety and psychosocial illness. A significant inverse correlation with life satisfaction was observed. Women exhibited greater fear of ageing than men.
Park [46]	2024	Positive Ageing Scale (PAS)	501 participants; mean age: 71.6 (6.4); 73% female. Inclusion criteria: age ≥ 60 years, self-reported fluent in English, resident of the UK.	Psychological	The results suggested that a unidimensional solution fits the data well, with the positive ageing factor adequately loading on 8 items, and that the solution exhibits factorial invariance between young-old and old participants (i.e. ≥75 years). Total PAS scores positively correlate with general health, well-being and cognitive functioning.
Ge [47]	2025	Health Resilience Scale (HRS)	650 participants (350 exploratory factor analysis and 300 confirmatory factor analysis); age repartition: 14.8% 21–30 years, 19.2% 31–40 years, 18.9% 41–50 years, 24.0% 51–60 years, 23.1% ≥61 years; 62.9% female. Inclusion criteria: age 21 years or older; Singapore citizen or permanent resident; and ability to independently read, comprehend and complete an English-language survey.	Psychological	The findings suggest that the 22-item HRS is a reliable and validated multi-dimensional tool for assessing individual health resilience. It offers valuable insights into the dynamic nature of health resilience, making it an effective scale for guiding initiatives to enhance health resilience and well-being in community settings. Future research is needed to further establish the psychometric properties of the HRS.
Mastela [48]	2023	Instrument to Assess Older Adults’ Satisfaction with Social Participation (IAPSI)	Phase 1: 102 participants; mean age: 87.3 (4.4); 74% female. Phase 2: 56 participants; mean age: not determined; 89.3% female. Inclusion criteria: Phase 1: adults of both sexes who were 60 years old or older and who were receiving care at a geriatrics and gerontology outpatient unit that provides public services in São Paulo. Phase 2: adults of both sexes who were 60 years or older and who had received care at the same outpatient unit in the first phase of the study. The inclusion criteria were participants who experienced chronic pain of different aetiologies with a minimum intensity of three on the numerical rating scale and were motivated to participate in this study, which required their involvement in assessments on two different days. Exclusion criteria: phase 1: older adults who presented with cognitive decline, as defined by a score in the Mini-Mental State Examination that is below the expected score for the individual’s level of education, or with neoplasm-related pain, or who had been hospitalised in the last three months, phase 2: those who presented with cognitive decline, as defined by a score on the Mini-Mental State Examination, or with neoplasm-related pain or those who had been hospitalised in the last 3 months.	Psychological, Vitality	A practical instrument has been developed with appropriate psychometric properties to assess older adults’ satisfaction with their social participation.
Farina [49]	2023	Fear and Avoidance of Memory Loss scale	813 participants. Two cohorts: USA: 513 participants; mean age: 66.3 (7.2); 68% female. International: 230 participants; mean age: 61.3 (5.2); 53% female. Inclusion criteria: being aged ≥55 years, being able and willing to provide informed consent, being able to read and write in English and having access to the internet for completion of study measures. Exclusion criteria: having a diagnosis of mild cognitive impairment, Alzheimer’s disease (AD) or other dementia by a healthcare provider, having an unstable medical condition (characterised as hospitalisation in the last 6 weeks or repeated emergency room visits), having inadequate vision or hearing to interact with study material or having a current substance use disorder.	Cognition, Psychological	Two subscales have been identified: fear and avoidance, which yielded strong psychometric validity. Higher fear was associated with memory failures and sleep disturbance. Higher avoidance was associated with memory failures, poorer verbal memory, reduced social functioning and quality of life.
Lim [50]	2022	Falls Health Literacy Scale (FHLS)	Phase 1: 144 participants; ≥18 years; median age 43 years (interquartile range, IQR = 32–53) Phase 2: 227 participants; community-living people aged ≥65 years; mean age: 75 (7) years. Inclusion criteria: Phase 1: aged >18 years, living in the community, speaking English; Phase 2: aged ≥65 years, living in the community, having no major cognitive, affective or physical problems, speaking English.	Locomotor, Cognition	The novel, context-specific FHLS displayed good construct validity and reliability. The FHLS holds promise as a screening tool to differentiate individuals with varying levels of fall-related health literacy, potentially helping guide fall-prevention interventions.
Deng [51]	2023	Physical Body Experiences Questionnaire simplified for Active ageing (PBE-QAG)	566 participants. —Part 1: 133 older adults without stroke; mean age: 70.3 (4.8); 58% female. —Part 2: 530 adults without stroke; mean age: 50.1 (16.8); 64% female. —Part 3: 36 with chronic stroke resulting in left or right hemiplegia or hemiparesis of the upper limb, mean age: 58.0 (12.9); 42% female. Inclusion criteria: overall: participants between 18 and 99 years old. Part 3: adults between 18 and 99 years; medically stable with an ischaemic or hemorrhagic stroke and resulting left or right hemiplegia, longer than 6 months from stroke-onset; able to sign consent, able to hear, read and comprehend instructions given during the study; and fluent in English. Exclusion criteria: overall: pregnant women and adults who did not speak English. Parts 1 & 2: adults with chronic stroke.	Locomotor, Psychological	PBE-QAG demonstrated good item and person fit, but the targeting is off. Therefore, the current version of PBE-QAG is not recommended for use in community-dwelling adults. The analyses of community-dwelling older adults and the larger group of adults revealed that rescoring and item deletion were required. Further validation of PBE-QAG by adding more difficult items is encouraged. Evaluating the PBE-QAG in a larger group of adults with stroke is also recommended.
Zhang [52]	2023	Disability Index (DI)	12 559 participants; mean age: 84.3 (11.2); 54.0% women. Inclusion criteria: data come from the Chinese Longitudinal Healthy Longevity Survey (CLHLS) and age > 65 years. Exclusion criteria: participants with missing data.	Locomotor, Cognition	The DI is a reliable and valid instrument to assess disability status among older adults.
Gunathilaka [53]	2023	Quality of Work Life Scale–Elderly (QOWLS-E)	250 participants; age range: 44% 60–64 years, 27% 65–70 years, 20% 71–74 years, 9% 75+; 36% female. Inclusion criteria: elderly worker (a worker aged 60 years or above, who had been working for the last 6 months, for a minimum of 20 hours per week at the time of recruiting for the study) Exclusion criteria: casual workers and seasonal workers	Locomotor, Psychological	A 35-item multidimensional scale (QOWLS-E), comprising nine domains, has demonstrated good construct validity and reliability.
Solomon [54]	2023	Risk score for clinically important declines in health and function (no official name)	3619 participants from two US cohorts; mean age: SWAN cohort 54.8 years and WHI cohort 54.9 years; 100% women. Inclusion criteria: women aged 42–52 years, women with less than 9 years between measurements were excluded.	Locomotor, Psychological, Vitality	It is a composite score calculated from several baseline characteristics to identify individuals at higher risk of future decline. It is a predictive index used to estimate the likelihood that a person will experience a significant deterioration in physical health or functioning over time.
Cella [55]	2022	Abbreviated Form of the Multidimensional Prognostic Index (BRIEF-MPI)	110 participants; mean age: 83.2 (6.9) years; 51.8% women. Inclusion criteria: age > 65 years; capacity to understand the project. Exclusion criteria: older people who did not want to participate in the project.	Locomotor, Psychological, Vitality	BRIEF-MPI showed good agreement with the full/standard version of the MPI, making this tool ideal for screening multidimensional frailty in older adults.
O’Brien [56]	2024	Frailty Index (FI)	4149 participants; mean age: 61.2 (95% CI: 60.9–61.4); 58.5% female. Inclusion criteria: age between 45 and 79, with or at risk for symptomatic femoral-tibial knee osteoarthritis. Exclusion criteria: (i) inflammatory arthritis; (ii) contraindication to 3T MRI; (iii) bilateral end-stage knee osteoarthritis.	Locomotor, Psychological, Vitality	The Frailty Index was developed using data from participants in the Osteoarthritis Initiative (OAI), and researchers tested whether it could predict death from any cause among these participants during the study period. The OAI is a large longitudinal research study and database used to investigate osteoarthritis and ageing-related outcomes related to all-cause mortality.
Nahar-van Venrooij [57]	2025	PH22 (Positive Health 22)	2457 participants; mean age: 53.3(17.8); 54% female; 39.9% high level of education; 39.8% visited a medical specialist at the hospital, psychiatrist, psychologist or psychotherapist in the last 12 months. Inclusion criteria: Dutch speaking; >18 years old.	Locomotor, Cognition, Psychological, Vitality	The PH22 demonstrated good psychometric properties, including a stable factor structure and evidence of construct validity. It showed an approximately normal distribution, with slightly more outliers at lower values and a stronger clustering of scores around the mean, particularly for the ‘Daily life management’ factor.
Lin [58]	2023	Healthy Brain Ageing–Functional Assessment Questionnaire (HBA-FAQ)	503 participants (comprising 24 healthy control participants, 111 with subjective cognitive decline, 312 with mild cognitive impairment and 56 with dementia, which comprised mostly those with AD); mean age: 67.6 (8.5) years; 60% female Inclusion criteria: aged ≥50 years with new onset mood or cognitive complaints. Exclusion criteria: intellectual disability or insufficient English proficiency for standardised neuropsychological assessment, Mini-Mental State Examination (MMSE) score < 20, history of nonaffective psychiatric disorder (e.g. schizophrenia), history of stroke, head injury with loss of consciousness >30 minutes or other neurological disorder (e.g. epilepsy), current substance dependence or abuse.	Locomotor, Cognition, Psychological, Vitality	The HBA-FAQ is a valid and reliable self-report tool that provides an efficient and sensitive approach to identifying subtle changes in daily functioning among older people at risk of dementia.
Lee [59]	2022	Centre of Excellence on Longevity Self-AdMinistered (CESAM) Questionnaire	120 participants; mean age: 79.7 (6.2) years; 62.5% female. Inclusion criteria: participants aged 65 and over attending an outpatient geriatric clinic, who understood English or Mandarin. Exclusion criteria: individuals who had severe neurological, cognitive or motor deficits.	Locomotor, Cognition, Psychological, Vitality.	The study validates the CESAM questionnaire as an online self-reported tool for identifying frailty. The CESAM questionnaire demonstrated excellent diagnostic performance for frailty using the Frailty Index (FI) and Clinical Frailty Scale (CFS).
Sarwar [60]	2022	Retrospective electronic frailty index (REFI)	2588 participants; mean age: 86.78 (10.1); 48.2% female. Inclusion criteria: electronic health records of residents admitted to Royal Freemasons Benevolent Institution (RFBI) from 1 January 2010 to 1 January 2021, aged 65 or over and with a stay period of over 6 months at residential aged care homes. Exclusion criteria: residents with missing data sources for more than four nonchronic and acute diseases and frailty deficits.	Locomotor, Cognition, Psychological, Vitality, Sensory	A novel REFI was developed using the routine data recorded at Residential Aged Care facilities (RAC). The utility of the REFI was demonstrated using data acquired from RFBI (26 RACs), and it has the potential to be adopted in national and international standards.
Hershkowitz Sikron [61]	2024	Meuhedet Electronic Frailty Index (MEFI)	120,986 participants; mean age: 73.9 (7.0) years; 54.3% women. Inclusion criteria: aged 65 years and over. Exclusion criteria: participants who left the Israeli Meuhedet Health Maintenance Organization in 2023.	Locomotor, Cognition, Psychological, Vitality, Sensory	The Electronic Frailty Index version created here is valid in predicting mortality or hospitalisation.

**Table 2:** Instruments extracted from Lopez *et al*.

Authors	Instrument name	IC domain
Guidici [13, 62]	Free and Cued Selective Reminding Test (FCRST)	Cognition
Giudici [13, 62] Huang [63]	Digit and Symbol Substitution Test (DSST)	Cognition
Huang [63]	Trail Making Test (TMT)—parts A and B	Cognition
Huang [63]	Wechsler Memory Scale (WMS)—Logical Memory I and II	Cognition
Lu [64]	Cantonese Chinese Montreal Cognitive Assessment version (MOCA)	Cognition
Brodaty [65]	General Practitioner Assessment of Cognition (GPCOG)	Cognition
Zhao [66] Zeng [67]	Tinetti	Locomotor
Giudici [13, 62] Gutierrez-Robledo [12] Huang [63, 68, 69] Locquet [9] Lu [64] Ma [70, 71] Ramirez-Velez [72] Yeung [73] Yu [74] Zeng [67] Gonzalez-Bautista [75, 76] Liu [77, 78]	Geriatric Depression Scale (GDS)	Psychological
Charles [79]	EUROQOL-5D	Psychological
Prince [80]	EURO-D depression scale	Psychological
Huang [63]	Generalized Anxiety Disorder (GAD-7)	Psychological
Yu [74]	Comprehensive Frailty Assessment Instrument (CFAI)—one item	Psychological
Charles [79]	Self-reported Strawbridge questionnaire	Sensory
Yeung [73] Yu [74, 81]	Frisby Stereo Test	Sensory
Gonzalez-Bautista [75, 76]	Hearing Handicap Inventory for the Elderly Screening (HHIE-S)	Sensory

The 46 studies comprised 258 383 participants, predominantly adults aged ≥60 years. Mean participant age exceeded 60 years in 78.7% of studies (range: 32.7–87.3 years), and sample sizes ranged from 20 to 120 986 participants, with 15 studies enrolling >1000 participants.

Thirty-two studies assessed a single IC domain, whereas 14 evaluated multidomain tools; only two included all five IC domains. Psychological capacity was the most frequently assessed domain (*n* = 24), followed by locomotion (*n* = 19), cognition (*n* = 15), vitality (*n* = 14) and sensory capacity (*n* = 2). Sensory capacity was never assessed as a standalone domain. Only one study was conducted exclusively in women.

Among single-domain tools, seven studies evaluated cognitive instruments, including TEUMI, BASIC-Q, MMQ-9, SHARE-cog, the Menu Task and a Risk Prediction Nomogram. Seven locomotor studies assessed handgrip strength, eSPPB, BPAAT, CTWT, SAB-M, sedentary behaviour algorithms and a fall prediction score. Five studies investigated vitality tools, including IPAB, T-SNAQ, NEI, AUTOMAL and a multimorbidity index. Thirteen studies evaluated psychological instruments such as the RSOA, PESOP, DA3, ECMES, BASIT-ER, SMILE, U-SIRS-13, Social Frailty Index, F-SOZU K-6, GEFAS, HRS and the Positive Ageing Scale.

Six instruments assessed two IC domains, whereas eight assessed three or more domains. Among the latter, PH22, HBA-FAQ and CESAM evaluated four domains, while REFI and MEFI covered all five IC domains.

Table 3 summarises the characteristics of all identified tools, including those reported by López-Ortiz *et al*. [8]. Based on these characteristics, psychometric properties and their clinical experience, MIPAG experts evaluated each tool for scientific validity, clinical relevance, clinical usability, patient self-assessment and suitability for adults aged ≤60 years. Mean scores are presented in Appendix 3.

**Table 3:** Overview of assessment tools for IC domains in adults (including tools in Lopez-Ortiz *et al*. review).

Name	Administration mode	Items (*n*)	Key characteristics	Scoring and interpretation
Cognition (12)				
Medication Literacy Test for Older Adults–Brazilian Portuguese (TELUMI)	Self-administered questionnaire	29 items (8 scenarios)	Evaluates the ability to understand and manage medication instructions.	≤10 points: Inadequate medication literacy; 11–20 points: Medium medication literacy; ≥21 points: Adequate medication literacy
Risk Prediction Nomogram	Predictive model	Multiple variables	Estimates the risk of cognitive decline.	Higher score = higher predicted risk
Brief Assessment of Impaired Cognition (BASIC)	Mixed: self, informant, tasks	3 parts (~10–15 minutes)	Combines subjective and objective cognition assessment.	<20 = possible cognitive impairment
Multifactorial Memory Questionnaire – short form (MMQ-9)	Self-administered	9 items	Assesses memory complaints, satisfaction and strategy use.	No universal cut-off
Cognitive Screening Instrument for the Survey of Health, Ageing and Retirement (SHARE-COG)	Survey-based	5–6 tasks	Used in large population studies (SHARE).	Lower scores = impairment (country-specific)
Menu Task	Performance task	Functional activity	Assesses executive function, planning and working memory.	Poor performance = dysfunction
Free and Cued Selective Reminding Test (FCRST)	Performance	16 items	Episodic memory	The Free and Cued Selective Reminding Test is a neuropsychological test used to evaluate episodic memory, particularly to help detect conditions such as Alzheimer’s disease. A total score < 46 means the person recalled fewer words than expected and may show signs of memory impairment.
Digit and Symbol Substitution test (DSST)	Performance	90–120 symbols	Processing speed	A lower DSST score means the person completed fewer correct substitutions, indicating slower processing speed and weaker cognitive performance. 70 correct answers: good performance; 40 correct answers: poor performance
Trail Making Test (TMT) A/B	Performance	2 tasks	Attention/executive	In the Trail Making Test (TMT), performance is measured by the time (in seconds) needed to complete the task. Therefore, longer times indicate worse cognitive performance. Task A > 78 s: Slower performance, possible impairment in attention or processing speed; task B > 273 s: poor performance, possible impairment in executive function
Wechsler Memory Scale (WMS) Logical Memory I/II	Performance	2 stories	Episodic recall	<1–1.5 SD indicates mild weakness in memory performance relative to peers, and >−2 SD indicates clearly impaired memory performance.
Cantonese Chinese Montreal Cognitive Assessment (MOCA)	Performance	30 items	Cognitive screen	<22–23 suggests a possible cognitive impairment
General practitioner Assessment of Cognition (GPCOG)	Mixed	12 items	Cognitive screen	If the patient scores 4 or less, this strongly suggests possible cognitive impairment or dementia. If the informant score is 3 or lower, it suggests a possible cognitive decline reported by someone close to the patient.
Brief Assessment of Impaired Cognition Questionnaire (BASIC-Q)	Informant-based questionnaire (typically administered by a clinician or researcher)	10 items	A brief cognitive screening tool designed to detect cognitive impairment and possible dementia. It combines questions about the individual’s current cognitive functioning, changes over time and functional abilities in everyday life.	Scores range from 0 to 10. A score ≤ 7 generally indicates possible cognitive impairment and suggests the need for further clinical assessment. Higher scores indicate better mental functioning.
Locomotor (9)				
Hand Grip Strength (HGS) measured with an aneroid sphygmomanometer	Performance test (dynamometer)	1 measure	Assesses upper-body muscle strength.	<27 kg (men) or <16 kg (women) indicates a possible sarcopenia or physical frailty, and further evaluation may be recommended.
electronic Short Physical Performance Battery (eSPPB)	Performance-based	3 tasks (balance, gait, chair stand)	Objective mobility assessment.	The electronic Short Physical Performance Battery (eSPPB) yields a total score ranging from 0 to 12 points that reflects lower-extremity physical function. 0–12 score. A score of 6 or less indicates high disability risk, meaning the person has reduced lower-limb function and mobility
Brief Physical Activity Assessment Tool (BPAAT)	Self-report	2–3 items	Rapid screen for physical activity level.	Higher BPAAT scores indicate greater physical activity; a score ≥ 4 suggests adequate activity, while ≤3 indicates insufficient activity.
Novel score for predicting 1-Year Falls	Predictive model	Composite	Estimates the risk of falls over the next 12 months.	The higher the score, the greater the likelihood that the person will experience a fall in the next 12 months.
Self-Assessment Burden Scale—Motor (SAB-M)	Self-administered	Short scale	Assesses burden of motor difficulty.	The SAB-M measures how strongly motor impairments affect a person’s daily activities, with higher scores indicating greater motor-related burden.
Circular Tandem Walk Test (CTWT)	Performance test	1 task	Assesses balance/coordination with circular walking.	The CTWT measures dynamic balance during tandem walking; difficulty maintaining the heel-to-toe path or frequent errors suggests impaired balance and increased risk of falls. Being unable to complete the test indicates a significant balance impairment and possible fall risk.
Algorithm for the Measurement of Sedentary Behaviour	Accelerometer-based	Continuous	Objective sedentary time assessment.	The algorithm estimates how long a person spends sitting or inactive; higher values indicate more sedentary behaviour and potentially greater health risk. High sedentary time is associated with increased risk of frailty. ≥ 8 hours/day: high sedentary behaviour (associated with higher frailty risk)
Tinetti	Performance	16 items	Balance/gait/falls	A score < 19 indicates a high risk of falling.
Vitality (5)				
Social Ecological Model – based Inventory of Physical Activity barriers Scale (IPAB)	Self-administered questionnaire	27 items	Assesses multilevel barriers to physical activity among adults 50+, using the Social Ecological Model.	The IPAB measures the number of barriers a person perceives to physical activity; higher scores indicate more barriers and a potentially lower likelihood of engaging in physical activity. Typically, there is no universal clinical cut-off; scores are used to compare barrier levels or identify the most important obstacles to physical activity.
Telephone administration of the Simplified Nutritional Appetite (T-SNAQ)	Telephone self-report	4 items	Appetite/nutrition risk screen.	A score < 14 suggests that the person has reduced appetite and is at risk of losing ≥5% of their body weight within the next 6 months.
Nutrition Equity Index (NEI)	Composite index	Multidomain	Evaluates fairness/equity of nutrition resources.	The NEI indicates how equitable access to adequate nutrition is; higher scores suggest better access to sufficient and healthy food, while lower scores suggest greater nutrition-related inequality.
AutoMal (automated malnutrition screening model)	EHR-derived model	BMI + weight loss	AI-based malnutrition screening.	AutoMal estimates the risk of malnutrition; higher scores indicate a higher predicted risk and the need for further nutritional assessment. It classifies individuals as at risk of malnutrition when the predicted probability is ≥0.37, and the model shows good discrimination (AUC ≈ 0.84).
Novel Multimorbidity Index	Clinical index	Chronic conditions	Assesses disease burden.	A higher Novel Multimorbidity Index score indicates that a person has more chronic diseases and a greater overall health burden. A higher NMI indicates greater multimorbidity, which is generally associated with lower vitality and a higher risk of functional decline.
Psychological (15)				
Psychological Empowerment Scale for Older People (PESOP)	Self-administered	Multiple items	Assesses autonomy and empowerment.	Higher PESOP scores indicate greater psychological empowerment among older adults, reflecting stronger feelings of autonomy, control and self-efficacy. There is usually no fixed clinical cut-off; scores are typically interpreted continuously or compared across groups.
Age Causes Illness Scale	Self-administered	7 items	Measures belief that ageing itself causes illness (self-directed ageism/health beliefs)	Higher scores indicate stronger beliefs that ageing inevitably causes illness, while lower scores reflect more positive or neutral beliefs about ageing and health. The scale typically does not have a strict clinical cut-off; scores are usually analysed continuously or compared across groups.
Resilience Scale for Older Adults (RSOA)	Self-administered	Multiple items	Measures psychological resilience.	Higher RSOA scores indicate greater resilience and better ability to cope with stress or adversity in later life. There is usually no universal cut-off; scores are interpreted continuously or compared between groups.
Depression, Anxiety, and Apathy scale (DA3)	Self-administered	3 subscales	Screens depression, anxiety, apathy.	Higher DA3 scores indicate more severe symptoms of depression, anxiety and apathy. Many studies do not use a universal clinical cut-off; instead, scores are used to compare symptom levels or to identify higher levels of psychological distress.
Emoji Current Mood and Experience Scale (ECMES)	Self-administered	Emoji-based	Captures emotional states.	Higher ECMES scores indicate a more positive current mood and emotional experience, while lower scores indicate a more negative emotional state. The scale usually does not have a fixed clinical cut-off; it is mainly used to monitor mood states or compare emotional experiences across individuals or time points.
Basel version of the Awareness of Social Inference Test–Emotion Recognition (BASIT-ER)	Self-administrated	Extended version compared to the original BASIT; includes more items covering frequency, quality and satisfaction with social interactions >15 items	Assesses perceived social connectedness and the burden of reduced interaction. Captures both quantitative aspects (how often social contact occurs) and qualitative aspects (meaningfulness, satisfaction)	Higher BASIT-ER scores indicate better ability to recognise emotions and interpret social signals. Most studies do not use a strict clinical cut-off; results are usually compared with normative data or group averages.
Senior Meaning in Life Evaluation (SMILE)	Self-administered	Variable	Assesses meaning and purpose.	Higher SMILE scores indicate greater perceived meaning in life among older adults. The SMILE does not usually have a fixed clinical cut-off; scores are interpreted continuously or compared between individuals or groups.
Upstream Social Interaction Risk Scale (U-SIRS-13)	Self-administrated	13 items	Capture reduced self-awareness in older adults	Higher U-SIRS-13 scores indicate a greater risk of limited social interaction or social isolation. The scale usually does not have a universal clinical cut-off; scores are often used continuously or compared between groups to identify higher social risk.
Social Frailty Index	Questionnaire	5–8 items	Measures social vulnerability.	Higher Social Frailty Index scores indicate greater social vulnerability and reduced social engagement or support. ≥2: social frailty
Perceived Social Support Questionnaire (F-SOZU K-6)	Self-administered	6 items	Assesses perceived social support.	Higher F-SOZU K-6 scores indicate greater perceived social support. The scale generally does not have a universal clinical cut-off; scores are typically interpreted continuously or compared between groups.
Gerascophobia or Excessive fear of Ageing Scale (GEFAS)	Self-administered	Varies	GEFAS: fear of ageing; HRS: resilience.	Higher GEFAS scores indicate greater fear or anxiety about ageing (gerascophobia). It does not use a strict clinical cut-off; scores are usually interpreted continuously or compared between groups.
Positive Ageing Scale (PAS)	Self-administered	Multiple items	Assesses positive attitudes towards ageing.	Higher PAS scores indicate more positive perceptions and attitudes towards ageing. The PAS usually does not have a universal clinical cut-off; scores are interpreted continuously or compared between groups.
Geriatric depression scale (GDS-15)	Self-administered	15 items	Depression	Higher GDS scores indicate greater depressive symptom severity. 0–4: Normal (no depression); 5–8: Mild depressive symptoms; 9–11: Moderate depression; 12–15: severe depression
Euro-D depression scale	Self-administered	12 items	Depression	A score ≥ 4 indicates clinically relevant depressive symptoms and suggests a possible case of depression that may need further evaluation
General Anxiety Disorder (GAD-7)	Self-administered	7 items	Anxiety	Score ≥ 5: presence of mild anxiety symptoms Score ≥ 10: moderate anxiety, often considered the clinical cut-off for possible generalised anxiety disorder Score ≥ 15: severe anxiety symptoms
Health Resilience Scale (HRS)	Self-administered questionnaire (or interviewer-administered for older adults with limitations)	20 items	Measures psychological resilience, focusing on coping, optimism, emotional regulation and adaptive capacity in older people	Higher scores: greater psychological health resilience Lower scores: reduced resilience/higher vulnerability
Sensory (2)				
Frisby Stereo Test	Performance	Plates	Stereoacuity	The Frisby Stereo Test is used to measure stereopsis, the ability to perceive depth (3-D vision) with both eyes. Smaller stereoacuity thresholds indicate better binocular depth perception, while larger thresholds indicate poorer stereoscopic vision. ≤ 40–60 arcsec: excellent depth perception
Hearing Handicap Inventory for the Elderly Screening (HHIE-S)	Self-admin	10 items	Hearing handicap	Higher HHIE-S scores indicate greater perceived hearing handicap; scores ≥10 suggest possible hearing problems requiring further assessment.
Combined/Multidomain Tools (9)				
Self-reported Strawbridge questionnaire	Self-administered	4–6 items	Subjective sensory impairment	The Self-reported Strawbridge questionnaire (often called the Strawbridge frailty or functional impairment questionnaire) is used to assess frailty and functional difficulties in older adults based on self-reported symptoms and limitations. Frailty is indicated when ≥2 functional domains (physical, cognitive, sensory or nutritional) show reported problems.
Quality of Work Life Scale-Elderly (QOWLS-E)	Self-administered	Multiple items	Assesses the quality of work life/roles in the old people.	Higher QOWLS-E scores indicate better perceived quality of work life in older adults. The scale generally does not have a universal cut-off; scores are interpreted continuously or compared across individuals or groups.
Physical Body Experiences Questionnaire simplified for Active ageing PBE-QAG	Self-administered	Simplified items	Assesses embodiment/body awareness.	Lower PBE-QAG scores indicate better body awareness and more positive body experiences during movement. The scale usually does not have a fixed clinical cut-off; scores are interpreted continuously or compared between groups.
Instrument to Access Older Adults’ Social Participation (IAPSI)	Self-administered questionnaire/structured interview	30 items	Covers multiple dimensions: frequency of participation, diversity of activities, autonomy in participation and satisfaction.	No universal cut-off; Higher scores = greater and more diverse social participation; Lower scores = risk of social isolation/restricted participation
Fall Health Literacy Scale (FHLS)	Self‑report questionnaire plus an objective knowledge test	25-item subjective scale, measuring self-reported fall-related health literacy;14-item objective scale, testing factual knowledge related to falls prevention	The subjective part assesses individuals’ perceived understanding of fall prevention, general health, staying active and seeking advice/services; The objective section provides a test-based measure where respondents answer multiple-choice questions tied to fall prevention scenarios and knowledge	No universal cut-offs. Higher subjective scores suggest better perceived health literacy; higher objective scores reflect stronger knowledge about falls prevention.
Fear and Avoidance of Memory Loss Scale	Self-administered	Multiple items	Assesses fear-driven avoidance behaviour.	Higher FAMLS scores indicate greater fear and avoidance related to perceived memory loss. The scale typically does not have a universal clinical cut-off; scores are interpreted continuously or compared across individuals or groups.
Positive Health 22 (PH22)	Self/informant -administered	22 items	Assesses positive health resources.	Higher PH22 scores indicate better perceived positive health and functioning across multiple health domains. The PH22 generally does not have a strict clinical cut-off. Scores are typically interpreted continuously or compared across domains or groups.
Retrospective electronic Frailty Index (REFI)	EHR-derived index	36 deficits	Frailty index from records.	Higher REFI scores indicate greater frailty due to the accumulation of multiple health deficits. > 0.30: Severe frailty
Health Brain Ageing – Functional Assessment Questionnaire HBA-FAQ	Self/informant -administered	~10–15 items	Assesses functional capacity in multiple domains.	Higher HBA-FAQ scores indicate greater functional impairment in daily activities related to cognitive functioning. Some studies use thresholds to indicate functional impairment, but the scale is often interpreted continuously or compared across groups.
Centre for Excellence on Longevity Self-Administered (CESAM) questionnaire	Self-administered	Multidomain	Assesses physical, cognitive, social and psychological.	Higher CESAM scores indicate a more favourable healthy ageing profile and better longevity-related health behaviours. The CESAM questionnaire usually does not have a strict clinical cut-off; scores are typically interpreted continuously or compared between groups.
Meuhedet Electronic Frailty Index (MEFI)	EHR-derived	Automated	Electronic frailty index (Israel).	The Meuhedet Electronic Frailty Index (MEFI) is a measure used to identify frailty in older adults, derived from electronic health record data. Higher MEFI scores indicate greater frailty, as measured by accumulated health deficits in electronic health records. Frailty index >0.30: severe frailty
EUROQOL-5D	Self-administered	5 + VAS	QoL	The EQ-5D Index (0–1) summarises a person’s overall health-related quality of life, where 1 = perfect health and 0 = death-equivalent health.
Comprehensive Frailty Assessment Instrument (CFAI single item)	Self-report	1	Frailty perception	The Comprehensive Frailty Assessment Instrument–single item (CFAI-SI) is a very brief self-report measure used to screen for overall frailty in older adults, consisting of a single global question. Higher CFAI-SI scores indicate greater self-perceived frailty in older adults. The single-item version usually has no strict universal cut-off; it is mainly used as a screening or quick self-assessment of frailty.
Abbreviated Form of the Multidimensional Prognostic Index (BRIEF MPI)	Clinician-administered (CGA-based)	~8 domains	Short version of the Multidimensional Prognostic Index using core CGA domains (function, cognition, comorbidities, nutrition, mobility, medications, social support) to estimate prognosis and vulnerability.	The Brief MPI is derived from the Multidimensional Prognostic Index, which is based on the Comprehensive Geriatric Assessment (CGA) used in Geriatrics. Higher Brief MPI scores indicate greater multidimensional frailty and a higher risk of adverse health outcomes. 0.67–1.00 High risk/severe frailty.
Disability Index (DI)	Interview/clinician-rated or self-report	Typically 10–14 items (ADL + IADL)	Measures limitations in basic and instrumental activities of daily living (e.g. bathing, dressing, shopping, managing medications/finances) to quantify functional impairment and care needs.	Higher Disability Index scores indicate greater functional disability and limitations in daily activities. 0 impaired items = independent; 1–2 impaired items = mild disability; ≥3 ADL impairments = severe disability/high care needs.
Frailty Index (FI)	Clinician-completed or automatically derived from records	≈30–70 deficits (symptoms, diseases, function, labs)	Cumulative deficit model in which frailty is expressed as the proportion of health deficits present across multiple systems; higher scores indicate greater frailty and vulnerability to adverse outcomes.	A higher FI index indicates greater frailty and higher vulnerability to adverse health outcomes. <0.10 = robust; 0.10–0.21 = pre‑frail; >0.21 = frail; >0.45 = very frail/very high risk.
Risk Score for Important Decline	Clinician-administered (often combined with simple performance or self-report items)	Commonly 4–10 items, depending on model	Predictive score estimating the probability of clinically meaningful decline in mobility or global function over time, typically combining age, comorbidities, prior functional decline and performance measures (e.g. gait speed/SPPB).	Cut‑offs model-dependent; the highest score categories generally = high risk. Frequently, gait speed <0.8 m/s or low SPPB (e.g. ≤8) is used as a high‑risk threshold.

Overall, the expert appraisal identified only a limited number of instruments that combined strong psychometric properties with high clinical usability. The GAD-7 and EUROQOL-5D achieved the highest overall score (25/25), reflecting excellent scientific validity, clinical relevance and ease of implementation. In cognition, the highest-rated tools were MMQ-9, the Risk Prediction Nomogram and BASIC. In locomotion and vitality, only the BPAAT and IPAB, respectively, scored above 20/25. Within the psychological domain, the highest-rated tools were the GAD-7 (25/25), F-SOZU K-6 (24/25) and the Geriatric Depression Scale (22.5/25). Sensory tools received lower ratings overall, with the HHIE-S outperforming the Frisby Stereo Test (18.5 vs. 10.5/25). Among multidomain instruments, the EUROQOL-5D, CESAM Questionnaire and Comprehensive Frailty Assessment Instrument (CFAI) obtained the highest overall scores. Self-administered questionnaires generally achieved higher usability scores than performance-based assessments or prediction models. Importantly, despite the inclusion of 61 assessment instruments, only a small proportion were considered suitable for adults younger than 60 years, reinforcing the current lack of tools capable of detecting early declines in IC before older age. Sensory and vitality domains also remained underrepresented, highlighting important gaps for future instrument development.

## Discussion

This scoping review provides an updated, comprehensive overview of validated tools for assessing IC. By identifying instruments published between 2022 and 2025 and supplementing them with tools from López-Ortiz *et al*.’s 2022 review [8], 61 tools were mapped across the five IC domains. We evaluated their scientific validity, clinical relevance and ease of use using an evaluation grid developed by MIPAG experts. Our expert evaluation specifically examined the suitability of existing IC assessment tools for adults younger than 60 years because our scoping review identified a lack of instruments designed for the early detection of IC decline. The aim was therefore to explore whether existing tools could potentially address this gap. Of course, the suitability and feasibility of these tools for adults over 80 years of age and for individuals with multimorbidity are also important considerations, particularly for routine geriatric practice and should be investigated. Because these aspects were not specifically evaluated, they represent a limitation of the present study and an important avenue for future research.

The findings reinforce the growing diversification of assessment strategies in ageing research and highlight significant gaps in measuring IC, especially among adults younger than 60 and in specific domains such as sensory and vitality. From a geriatric perspective, the clinical value of IC assessment lies not only in psychometric robustness but also in its ability to inform concrete clinical decisions, such as care planning, referral pathways, deprescribing strategies and targeted preventive interventions.

The most common tools identified focused on the psychological and locomotor domains, confirming patterns noted in earlier reviews. Tools such as the GAD-7 and various physical performance assessments demonstrated strong psychometric support and scored highly for clinical relevance and usability, according to the expert panel that evaluated them. Their prominence reflects both the extensive validation history in these areas and the established role of mood, anxiety and mobility measures in geriatric screening, frailty assessment and IC surveillance. Early identification of subclinical, potentially reversible declines is a core principle of geriatric medicine; therefore, IC instruments sensitive to subtle changes in mobility, cognition and vitality are especially relevant for preventive and prehabilitative strategies. The abundance of validated instruments for locomotor and psychological assessment supports the feasibility of incorporating these IC assessments into routine clinical care, especially in primary care settings, where quick, reliable and familiar tools are essential. In contrast, the vitality and sensory domains are still significantly underrepresented, with fewer tools identified and generally lower expert scores—particularly regarding patient usability and suitability for younger adults.

These gaps are consistent with previous observations that vitality, as defined by the WHO, is a multidimensional concept extending beyond nutrition and physical function to reflect overall physiological health. Consequently, current assessments rely on fragmented proxies, and validated tools for this IC domain remain limited. Similar gaps exist for sensory function, where the lack of validated, rapid and feasible assessment tools limits comprehensive IC evaluation despite the strong association between sensory decline, frailty, cognitive impairment and accelerated biological ageing. Early cognitive assessment before age 60 is also uncommon, representing a missed opportunity for prevention. Overall, IC assessment should support person-centred, goal-oriented care by focusing on functional reserve, patient priorities and quality of life rather than solely on deficit accumulation.

Overall, IC assessment instruments used varied widely, from domain-specific scales (e.g. Resilience Scale for Older Adults, Handgrip strength) to more integrative tools (e.g. CESAM, MEFI). Still, few provided a comprehensive evaluation of IC. Their feasibility in real-world geriatric settings also remains an important challenge. In very old or frail adults, routine implementation may be limited by cognitive impairment, sensory deficits (vision and hearing), fatigue, multimorbidity, reduced mobility, low health and digital literacy, administration time, the need for trained personnel or specialised equipment and, in some cases, reliance on proxy respondents. These factors should be carefully considered when selecting IC assessment tools for use in outpatient clinics, primary care or long-term care settings. Our results partially align with those of López-Ortiz *et al*. [8] and George *et al*. [7], who also reported a concentration of validated tools in the psychological and locomotor domains, alongside a scarcity of instruments suitable for vitality, sensory function or multidomain IC assessment. However, by extending the search timeframe to 2025 and including midlife adults alongside older populations, our review provides a more comprehensive, life-course-oriented mapping of available assessment tools. Despite increasing research interest, innovation in measurement for IC is progressing unevenly across domains and remains largely older-adult-centric. Furthermore, by incorporating expert scoring, we provide a pragmatic layer of interpretation missing from earlier reviews, enabling clinicians to weigh tool suitability beyond purely psychometric considerations.

A central goal of this review was to determine whether tools validated in younger populations (<60 years) exist, thereby supporting the shift towards early-life IC surveillance and the establishment of strategies for IC optimisation and decline prevention. Our findings reveal that most tools remain validated exclusively in older adults, with only a minority reporting psychometric testing in younger samples or across wide age ranges [25, 39, 45, 50, 51, 54, 57]. This underscores the need to develop and validate assessment instruments for use in middle-aged and younger adults, a life period during which IC trajectories begin to diverge and preventive interventions may be most effective.

Despite the heterogeneity, many of the identified instruments have clinical value. Tools such as the Disability Index or the Short Physical Performance Battery are practical for routine use. In contrast, psychosocial measures such as the Positive Ageing Scale provide insights into subjective well-being. More integrative tools (e.g. CESAM, MEFI) show promise for holistic assessment aligned with WHO’s IC framework. Wider adoption of such tools could facilitate early detection of decline, targeted interventions and promotion of positive ageing in both clinical and community settings.

**Figure 2:**
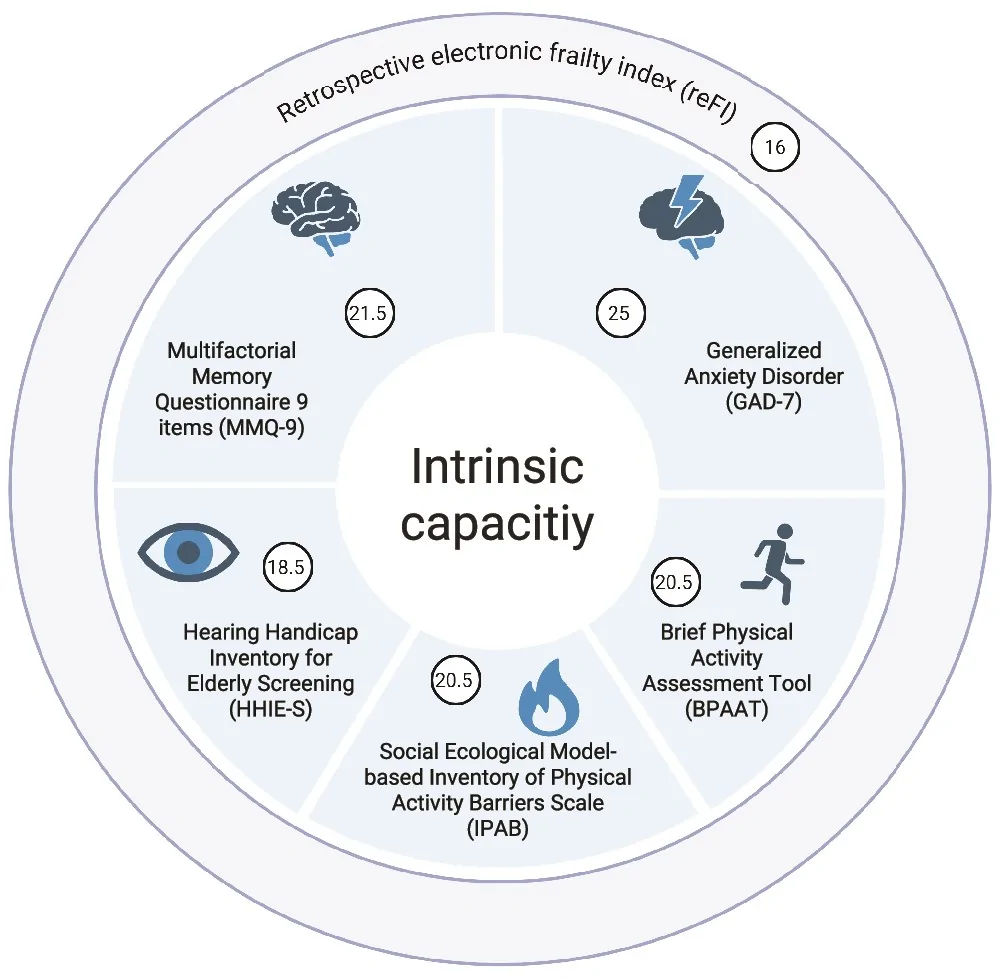
The top-ranked instruments to evaluate intrinsic capacities based on their scientific validity, clinical relevance, usability in clinical practice, usability by patients directly, and suitability for <60-year-old patients. The number in the circle corresponds to the total score, the sum of the scores for the five evaluation criteria. The maximum score is 25.

The expert scoring exercise revealed that patient usability and suitability for younger adults contributed most to the variability in scores. Tools with excellent scientific validity often performed poorly in these aspects (e.g. MOCA, WMS Logical Memory), suggesting that psychometric sophistication does not automatically translate into clinical practicality. This reinforces the notion that IC assessment tools must balance methodological rigour with real-world feasibility to be adopted in primary care, digital health platforms or community-based preventive programmes (Figure 2).

Instruments capable of assessing multiple IC domains simultaneously—such as the EUROQOL-5D or the CESAM questionnaire—achieved strong overall scores and may represent promising options for integrated IC assessment. However, few multidomain instruments explicitly map onto the IC framework or have undergone domain-specific validation, limiting their interpretability when used to guide IC-targeted interventions. Developing multidomain tools or IC-specific composites remains a vital area for research.

Several studies included in this review used frailty indices, such as the REFI and MEFI, within the context of IC assessment. However, these instruments should not be considered direct measures of IC. Although frailty and IC are closely related and often coexist in older adults, they represent conceptually distinct constructs. Frailty is a state of increased vulnerability resulting from the accumulation of health deficits and reflects a reduced ability to withstand physiological stressors. In contrast, according to the World Health Organization, intrinsic capacity refers to the composite of an individual’s physical and mental capacities at a given time, independent of environmental influences. Whereas frailty emphasises disease burden, deficit accumulation and vulnerability, IC focuses on the preservation and optimisation of functional capacities across its five domains. Consequently, frailty indices should not be regarded as surrogate measures of IC, as doing so may obscure the conceptual distinction between these constructs and introduce ambiguity into both research and clinical practice. Future research should therefore prioritise the development and validation of IC-specific assessment tools, including electronic health record–based approaches that remain aligned with the WHO conceptual framework by measuring capacity domains rather than disease burden or deficit accumulation.

A significant strength of this review is its updated evidence base, which captures studies published up to 2022, and the use of a comprehensive protocol that adheres to methodological standards for scoping reviews. Furthermore, we included only validated tools, ensuring the reliability and relevance of the synthesised evidence. Another strength is the use of a structured, multidisciplinary, expert-based evaluation system that captures the pragmatic dimensions of tool usability. Emphasis on lifespan applicability, reflecting the evolving WHO conceptualisation of IC as a continuum beginning in early adulthood, is also remarkable.

Nevertheless, certain limitations must be acknowledged. First, the 2022 cut-off year excludes earlier tools and studies, potentially missing foundational evidence. Second, the language restriction and exclusion of grey literature may have limited the diversity of instruments captured, especially those developed in non-English-speaking contexts. The expert scoring, while clinically informative, also raises concerns: first, it is inherently subjective and may have introduced variability, as reflected in large standard deviations. Second, a formal inter-rater reliability measure was not implemented and should be considered in future work. Third, the scoring system used equal weighting across criteria, though some dimensions (e.g. scientific validity) may warrant greater emphasis depending on context. Many tools lacked validation data in younger adults, limiting our ability to assess actual lifespan applicability. Fourth, our inclusion of tools from López-Ortiz *et al*. relied on previously reported evidence rather than independent re-extraction, which may introduce inconsistencies in data depth. Finally, although scoping reviews aim to map evidence rather than evaluate quality, the heterogeneous reporting of validation studies across tools made comparison challenging.

Finally, although most included studies involved participants with a mean age above 60 years, limited information was available on the specific factors affecting the usability of IC assessment tools in very old or frail populations. Cognitive, visual or hearing impairments, fatigue, multimorbidity, limited digital literacy, lengthy administration procedures and the potential need for proxy assistance may reduce feasibility in routine geriatric practice. Moreover, floor or ceiling effects may limit the ability of some instruments to detect meaningful differences or changes across the broad range of functional abilities observed in older adults. These aspects were not consistently assessed in the included studies and should be specifically investigated when validating IC assessment tools in adults over 80 years of age and in multimorbid or frail populations.

These findings highlight key priorities for research and clinical practice. A new multidimensional tool is needed to assess IC in younger adults before significant decline occurs, enabling timely preventive interventions. Further studies should also investigate sex-related differences in IC, as only one study addressed this issue. To support the development of such a tool, an expert consensus process should be planned to identify the most relevant assessment instruments and items. A standardised tool covering all five IC domains would improve comparability across studies, facilitate longitudinal monitoring and clinical decision-making and support a more proactive approach to healthy ageing.

This scoping review highlights substantial progress in measuring IC while revealing critical gaps that currently limit its full clinical and preventive potential. Although robust and feasible tools are available for the psychological and locomotor domains, validated instruments for vitality and sensory function remain scarce, and most assessments remain confined to older populations. Bridging these gaps is essential to shift IC from a predominantly descriptive construct to a truly actionable, life-course-oriented clinical framework. Our findings also highlight the need to evaluate the feasibility and measurement properties of brief, sensory-adapted and, where appropriate, proxy-assisted versions of IC assessment tools for older adults, particularly for the vitality and sensory domains. In addition, feasibility in real-world geriatric settings—including primary care, long-term care facilities and home care—remains insufficiently investigated and represents an important implementation gap. Developing multidomain, user-friendly tools applicable from midlife onward will be key to enabling early detection of decline, guiding personalised interventions and supporting proactive strategies for healthy and positive ageing across adulthood.

## Supplementary Material

Supplementary_material_afag274
