# Supplementary material for: Existing validated tools for assessing intrinsic capacity and its components within a positive ageing framework: a scoping review and structured appraisal by the Multidisciplinary International Positive Ageing Group
Source: Age Ageing. 2026 Sep 21;55(9):afag274. doi: 10.1093/ageing/afag274 (PMC13591483; doi:10.1093/ageing/afag274)
Supplement: Supplementary file 1 [file supplementary_material_afag274.zip › aa-26-1600-File007.docx]

Existing validated tools for assessing intrinsic capacity and its components within a positive ageing framework: A scoping review and structured appraisal by the Multidisciplinary International Positive Ageing Group (MIPAG)

Appendix 3

| EXPERT APPRAISAL SCORES OF IC ASSESSMENT TOOLS (N=61) | | | | | | |
| --- | --- | --- | --- | --- | --- | --- |
| Name(s) of the tool(s) | Scientific validity Mean (SD) | Clinical relevance  Mean (SD) | Usability in clinical practice Mean (SD) | Patient self-assessment usability Mean (SD) | Suitability for patients younger than 60 years  Mean (SD) | Total score |
| Cognitive domain (13) | | | | | | |
| Multifactorial Memory Questionnaire 9 items (MMQ-9) | 4.5 (0.7) | 4.5 (0.7) | 4.5 (0.7) | 3 (2.8) | 5 (0) | 21.5 (9.9) |
| Risk-prediction nomogram | 4 (0) | 4.5 (0.7) | 4 (0) | 3.5 (2.1) | 5 (0) | 21 (12.0) |
| Brief Assessment of Impaired Cognition (BASIC) | 4 (1.4) | 5 (0) | 5 (0) | 3.5 (0.7) | 3.5 (2.1) | 21 (14.1) |
| Cantonese Chinese Montreal Cognitive Assessment version (MOCA) | 5 (0) | 5 (0) | 5 (0) | 1 (0) | 4.5 (0.7) | 20.5 (0.7) |
| Wechsler Memory Scale (WMS) - Logical Memory I and II | 5 (0) | 4.5 (0.7) | 4 (0) | 1.5 (0.7) | 5 (0) | 20 (0) |
| Digit and Symbol Substitution Test (DSST) | 4 (1.4) | 4.5 (0.7) | 4 (1.4) | 2 (0) | 5 (0) | 19.5 (3.5) |
| Brief Assessment of Impaired Cognition– Questionnaire (BASIC-Q) | 3 (1.4) | 4.5 (0.7) | 5 (0) | 4 (0) | 3 (1.4) | 19.5 (12.0) |
| Cognitive Screening Instrument for the Survey of Health, Ageing and Retirement (SHARE-COG) | 4.5 (0.7) | 5 (0) | 4.5 (0.7) | 1 (0) | 4 (1.4) | 19 (12.0) |
| General Practitioner Assessment of Cognition (GPCOG) | 3.5 (0.7) | 4.5 (0.7) | 4 (1.4) | 2.5 (0.7) | 4 (1.4) | 18.5 (0.7) |
| Free and Cued Selective Reminding Test (FCRST) | 5 (0) | 4 (0) | 3.5 (0.7) | 1 (0) | 4.5 (0.7) | 18 (1.4) |
| Trail Making Test (TMT) - parts A and B | 3 (2.1) | 3.5 (2.1) | 4 (1.4) | 1.5 (0.7) | 5 (0) | 17.5 (4.9) |
| Menu Task | 4 (0) | 4.5 (0.7) | 3.5 (0.7) | 1 (0) | 4.5 (0.7) | 17.5 (9.2) |
| TELUMI (Medication Literacy Test for Older Adults - Brazilian Portuguese Acronym) | 3 (0) | 3.5 (0.7) | 3 (0) | 2 (0) | 4 (0) | 15.5 (7.8) |
| Locomotor (8) | | | | | | |
| Brief Physical Activity Assessment Tool | 3.5 (2.1) | 3.5 (0.7) | 4.5 (0.7) | 4.5 (0.7) | 4.5 (0.7) | 20.5 (9.9) |
| electronic Short Physical Performance Battery (eSPPB) | 5 (0) | 4.5 (0.7) | 4.5 (0.7) | 3 (1.4) | 2 (0) | 19 (13.4) |
| Tinetti | 5 (0) | 4.5 (0.7) | 4 (0) | 2 (0) | 2.5 (0.7) | 18 (1.4) |
| Novel score for predicting 1-year falls | 4.5 (0.7) | 4.5 (0.7) | 3.5 (0.7) | 2 (0) | 3 (1.4) | 17.5 (11.3) |
| Hand Grip Strength (HGS) measured with an aneroid sphygmomanometer | 4 (1.4) | 4 (0) | 4 (0) | 2 (0) | 2.5 (0.7) | 16.5 (8.5) |
| Circular tandem walk test (CTWT) | 4 (0) | 4 (0) | 4 (0) | 2.5 (0.7) | 2 (0) | 16.5 (10.6) |
| The Self-Assessment Burden Scale-Motor (SAB-M) | 4.5 (0.7) | 4 (0) | 4 (0) | 2 (1.4) | 1.5 (0.7) | 16 (8.5) |
| Algorithm for Measurement of Sedentary Behaviour | 3.5 (2.1) | 3.5 (0.7) | 2.5 (2.1) | 1.5 (0.7) | 2 (0) | 13 (4.9) |
| Vitality (5) | | | | | | |
| Social Ecological Model-based Inventory of Physical Activity Barriers Scale (IPAB) | 4 (0) | 4.5 (0.7) | 3 (0) | 4.5 (0.7) | 4.5 (0.7) | 20.5 (12.0) |
| Telephone administration of the Simplified Nutritional Appetite Questionnaire (T-SNAQ) | 4 (1.4) | 4.5 (0.7) | 4 (0) | 4 (1.4) | 2.5 (0.7) | 19 (12.7) |
| AUTOMAL (automated malnutrition screening model) | 3.5 (0.7) | 4.5 (0.7) | 4.5 (0.7) | 4.5 (0.7) | 1.5 (0.7) | 18.5 (12.0) |
| Nutrition Equity Index (NEI) | 3.5 (2.1) | 4 (1.4) | 3.5 (0.7) | 4 (1.4) | 2.5 (0.7) | 17.5 (12.7) |
| Novel multimorbidity index | 4 (0) | 4 (1.4) | 2.5 (0.7) | 1.5 (0.7) | 3 (1.4) | 15 (10.6) |
| Psychological (16) | | | | | | |
| Generalized Anxiety Disorder (GAD-7) | 5 (0) | 5 (0) | 5 (0) | 5 (0) | 5 (0) | 25 (0) |
| Perceived Social Support Questionnaire (F-SOZU K-6) - Brief form (6 items) | 5 (0) | 4.5 (0.7) | 4.5 (0.7) | 5 (0) | 5 (0) | 24 (13.4) |
| Geriatric Depression Scale (GDS) | 5 (0) | 4.5 (0.7) | 5 (0) | 4.5 (0.7) | 3.5 (2.1) | 22.5 (3.5) |
| Gerascophobia or Excessive Fear of Ageing Scale (GEFAS) | 4.5 (0.7) | 3.5 (0.7) | 4.5 (0.7) | 5 (0) | 5 (0) | 22.5 (12.0) |
| The Emoji Current Mood and Experience Scale (ECMES) | 4 (0) | 4 (1.4) | 5 (0) | 5 (0) | 4.5 (0.7) | 22.5 (12.7) |
| Depression, Anxiety, and Apathy (DA3) scale | 4.5 (0.7) | 4.5 (0.7) | 5 (0) | 4.5 (0.7) | 3.5 (0.7) | 22 (14.8) |
| Health Resilience Scale (HRS) | 5 (0) | 4.5 (0.7) | 3 (1.4) | 4 (1.4) | 5 (0) | 21.5 (12.0) |
| Social Frailty Index | 4 (1.4) | 4.5 (0.7) | 4.5 (0.7) | 4.5 (0.7) | 4 (1.4) | 21.5 (12.7) |
| Upstream Social Interaction Risk Scale (U-SIRS-13) | 4 (1.4) | 4 (0) | 4.5 (0.7) | 4 (1.4) | 4.5 (0.7) | 21 (12.7) |
| Positive Ageing Scale (PAS) | 5 (0) | 4 (1.4) | 3.5 (0.7) | 5 (0) | 3.5 (2.1) | 21 (14.8) |
| Euro-D depression scale | 5 (0) | 4.5 (0.7) | 3.5 (0.7) | 3 (0) | 3.5 (0.7) | 19.5 (0.7) |
| Age Causes Illness scale | 3.5 (0.7) | 2.5 (0.7) | 4 (1.4) | 5 (0) | 4.5 (0.7) | 19.5 (10.6) |
| Resilience Scale for Older Adults (RSOA) | 4.5 (0.7) | 4 (0) | 4 (0) | 3.5 (0.7) | 2.5 (0.7) | 18.5 (12.0) |
| Psychological Empowerment Scale for Older People (PESOP) | 4.5 (0.7) | 3 (0) | 4 (0) | 4.5 (0.7) | 2.5 (0.7) | 18.5 (12.7) |
| SMiLE (Senior Meaning in Life Evaluation) | 4 (0) | 3.5 (0.7) | 3 (1.4) | 3 (1.4) | 3 (1.4) | 16.5 (6.4) |
| The Basel Version of the Awareness of Social Inference Test-Emotion Recognition (BASIT-ER) | 3.5 (0.7) | 3 (0) | 1.5 (0.7) | 2 (1.4) | 4 (1.4) | 14 (8.5) |
| Sensory (2) | | | | | | |
| Hearing Handicap Inventory for the Elderly Screening (HHIE-S) | 3 (2.8) | 4 (1.4) | 3.5 (0.7) | 4.5 (0.7) | 3.5 (2.1) | 18.5 (7.8) |
| Frisby Stereo Test | 2 (1.4) | 3 (1.4) | 2 (0) | 1.5 (0.7) | 2 (0) | 10.5 (0.7) |
| Multidomain Instruments (17) | | | | | | |
| EUROQOL-5D | 5 (0) | 5 (0) | 5 (0) | 5 (0) | 5 (0) | 25 (0) |
| Centre of Excellence on Longevity Self-AdMinistered (CESAM) Questionnaire | 5 (0) | 5 (0) | 4.5 (0.7) | 5 (0) | 3 (2.8) | 22.5 (10.6) |
| Comprehensive Frailty Assessment Instrument (CFAI) - one item | 5 (0) | 4.5 (0.7) | 4.5 (0.7) | 4 (1.4) | 2.5 (2.1) | 20.5 (4.9) |
| PH22 (Positive Health 22) | 4 (1.4) | 4 (1.4) | 3 (0) | 4 (1.4) | 4.5 (0.7) | 19.5 (14.1) |
| Self-reported Strawbridge questionnaire | 3.5 (2.1) | 3.5 (2.1) | 3.5 (2.1) | 4.5 (0.7) | 3 (1.4) | 18 (8.5) |
| Physical Body Experiences Questionnaire simplified for Active ageing (PBE-QAG) | 4 (0) | 3 (0 | 3.5 (0.7) | 4.5 (0.7) | 3 (2.8) | 18 (7.8) |
| Frailty Index (FI) | 5 (0) | 4.5 (0.7) | 3.5 (0.7) | 1.5 (0.7) | 3.5 (0.7) | 18 (9.2) |
| Abbreviated Form of the Multidimensional Prognostic Index (BRIEF-MPI) | 5 (0) | 4.5 (0.7) | 4.5 (0.7) | 2 (1.4) | 1.5 (0.7) | 17.5 (9.9) |
| "Risk score for clinically important declines in health and function" (no official name) | 4 (0) | 3.5 (0.7) | 3.5 (0.7) | 2.5 (2.1) | 3.5 (2.1) | 17 (9.9) |
| Fear and Avoidance of Memory Loss scale | 3.5 (2.1) | 3.5 (0.7) | 3.5 (0.7) | 4 (0) | 2.5 (0.7) | 17 (12.7) |
| Healthy Brain Ageing – Functional Assessment Questionnaire (HBA-FAQ) | 4 (1.4) | 4 (0) | 2.5 (0.7) | 2.5 (0.7) | 3.5 (0.7) | 16.5 (9.9) |
| Falls Health Literacy Scale (FHLS) | 4 (1.4) | 3.5 (0.7) | 3 (0) | 4 (0) | 2 (0) | 16.5 (10.6) |
| Retrospective electronic frailty index (REFI) | 4.5 (0.7) | 4.5 (0.7) | 4 (1.4) | 1.5 (0.7) | 1.5 (0.7) | 16 (7.8) |
| Disability Index (DI) | 5 (0) | 4 (0) | 3.5 (0.7) | 1.5 (0.7) | 1.5 (0.7) | 15.5 (8.5) |
| Instrument to Assess Older Adults’ Social Participation (IAPSI) | 3.5 (0.7) | 3 (0) | 4 (1.4) | 3 (0) | 1.5 (0.7) | 15 (9.9) |
| Quality of Work Life Scale-Elderly (QOWLS-E) | 4 (0) | 3 (0) | 2.5 (0.7) | 2 (1.4) | 2.5 (2.1) | 14 (4.9) |
| Meuhedet Electronic Frailty Index (MEFI) | 4 (0) | 4 (1.4) | 2 (0) | 1.5 (0.7) | 1.5 (0.7) | 13 (5.7) |

SD: Standard Deviation
