# Supplementary material for: Existing validated tools for assessing intrinsic capacity and its components within a positive ageing framework: a scoping review and structured appraisal by the Multidisciplinary International Positive Ageing Group
Source: Age Ageing. 2026 Sep 21;55(9):afag274. doi: 10.1093/ageing/afag274 (PMC13591483; doi:10.1093/ageing/afag274)
Supplement: Supplementary file 1 [file supplementary_material_afag274.zip › aa-26-1600-File005.docx]

Existing validated tools for assessing intrinsic capacity and its components within a positive ageing framework: A scoping review and structured appraisal by the Multidisciplinary International Positive Ageing Group (MIPAG)

Date : update 7 mai 2025

Information sources

Medline (PubMed interface, 1946 onwards)

Embase (Elsevier interface, 1974 onwards)

CINAHL (EBSCOhost platform, 1931 onwards)

PsycINFO (Ovid platform, 1806 onwards)

| Bibliographic databases | |  | Concept | Search | n results |
| --- | --- | --- | --- | --- | --- |
| Medline (PubMed) | | #1 |  | "Patient Outcome Assessment"[Mesh] OR "Artificial Intelligence"[Mesh] OR "Machine Learning"[Mesh] OR "scor*"[Title/Abstract] OR "test*"[Title/Abstract] OR "assessment*"[Title/Abstract] OR "evaluation*"[Title/Abstract] OR "index"[Title/Abstract] OR "measure*"[Title/Abstract] OR "PROM"[Title/Abstract] OR "PROMs"[Title/Abstract] OR "PRO"[Title/Abstract] OR "PROs"[Title/Abstract] OR "patient outcome"[Title/Abstract:~3] OR "patient outcomes"[Title/Abstract:~3] OR "rating*"[Title/Abstract] OR "questionnaire*"[Title/Abstract] OR "survey*"[Title/Abstract] OR "scale*"[Title/Abstract] OR "instrument*"[Title/Abstract] OR "monitoring"[Title/Abstract] OR "indices"[Title/Abstract] OR "indicator*"[Title/Abstract] OR "clinical prediction model*"[Title/Abstract] OR "machine learning"[Title/Abstract] OR "artificial intelligence"[Title/Abstract] OR "deep learning"[Title/Abstract] OR "deep-learning"[Title/Abstract] | 12,790,833  results |
|  |  |  | AND |  |  |
|  |  |  |  | "validat*"[Title] OR "approv*"[Title] OR "demonstrat*"[Title] | 200,231  results |
|  |  |  | AND |  |  |
|  |  |  |  | "Geriatric Assessment"[Mesh] OR "Aged"[Mesh] OR "Aging"[Mesh] OR "aging"[Title/Abstract] OR "ageing"[Title/Abstract] OR "aged"[Title/Abstract] OR "elder"[Title/Abstract] OR "older"[Title/Abstract] OR "elderly"[Title/Abstract] OR "decline"[Title/Abstract] OR "senile"[Title/Abstract] OR "senility"[Title/Abstract] OR "senior"[Title/Abstract] OR "advanced age"[Title/Abstract] OR "geriatri*"[Title/Abstract] | 5,092,339  results |
|  | |  | AND |  |  |
|  | |  |  | "Quality of Life"[Mesh] OR "Healthy Aging"[Mesh] OR "Activities of Daily Living"[Mesh] OR "Independent Living"[Mesh] OR "Healthy Life Expectancy"[Mesh] OR "intrinsic capacit*"[Title/Abstract] OR "physical"[Title/Abstract] OR "well being"[Title/Abstract] OR "quality life"[Title/Abstract :~3] OR "qualities life"[Title/Abstract:~3] OR "QOL"[Title/Abstract] OR "functional ability"[Title/Abstract:~3] OR "functional abilities"[Title/Abstract:~3]OR "detection"[Title/Abstract] OR "prevention"[Title/Abstract] OR "frail*"[Title/Abstract] OR "abilit*"[Title/Abstract] OR "capacit*"[Title/Abstract] OR "disabilit*"[Title/Abstract] OR "disorder*"[Title/Abstract] OR "dysfunction*"[Title/Abstract] OR "deficienc*"[Title/Abstract] OR "impairment*"[Title/Abstract] OR "locomotor"[Title/Abstract] OR "locomotion"[Title/Abstract] OR "physiological"[Title/Abstract] OR "resilience"[Title/Abstract] OR "cogniti*"[Title/Abstract] OR "psycholog*"[Title/Abstract] OR "psychiatry"[Title/Abstract] OR "sensory"[Title/Abstract] OR "social"[Title/Abstract] OR "integration"[Title/Abstract] OR "vitality"[Title/Abstract] OR "fatigue"[Title/Abstract] OR "positive"[Title/Abstract] OR "healthy"[Title/Abstract] OR "successful"[Title/Abstract] OR "active"[Title/Abstract] OR "activity"[Title/Abstract]) | 14,132,718  results |
|  |  | | | | |
| PubMed (7 mai 2025) : 18,151 results  Si limite ≥ 2022 : 4,431 results  Pour tester : https://pubmed.ncbi.nlm.nih.gov/?term=longqueryfdb62c334da71d1a1254&filter=years.2022-2025 | | | | | |
|  |  | | | | |
| Embase (Elsevier) | |  |  | 'patient-reported outcome'/exp OR 'artificial intelligence'/syn OR 'machine learning'/syn OR 'scoring system'/syn OR 'deep learning'/syn OR (patient* NEAR/3 outcome*):ab,ti OR scor*:ab,ti OR test*:ab,ti OR assessment*:ab,ti OR evaluation*:ab,ti OR index:ab,ti OR measure*:ab,ti OR prom:ab,ti OR proms:ab,ti OR rating*:ab,ti OR questionnaire*:ab,ti OR survey*:ab,ti OR scale*:ab,ti OR instrument*:ab,ti OR monitoring:ab,ti OR indices:ab,ti OR indicator*:ab,ti OR 'clinical prediction model*':ab,ti | 16,931,863 results |
|  | |  | AND |  |  |
|  | |  |  | validat*:ti OR approval*:ti OR approved*:ti OR demonstrat*:ti | 260,060 results |
|  | |  | AND |  |  |
|  | |  |  | 'geriatric assessment'/syn OR 'aging'/de OR 'healthy aging'/syn OR aging:ab,ti OR ageing:ab,ti OR aged:ab,ti OR elder:ab,ti OR older:ab,ti OR elderly:ab,ti OR decline:ab,ti OR senile:ab,ti OR senility:ab,ti OR senior:ab,ti OR 'advanced age':ab,ti OR geriatri*:ab,ti | 2,876,671 results |
|  | |  | AND |  |  |
|  | |  |  | 'quality of life'/syn OR 'daily life activity'/syn OR 'independent living'/syn OR 'healthy life expectancy'/syn OR intrinsic*:ab,ti OR physical:ab,ti OR 'well being':ab,ti OR 'well-being':ab,ti OR (qualit* near/2 life):ab,ti OR qol:ab,ti OR (functional near/3 abilit*):ab,ti OR detection:ab,ti OR prevention:ab,ti OR frail*:ab,ti OR abilit*:ab,ti OR capacit*:ab,ti OR disabilit*:ab,ti OR disorder*:ab,ti OR dysfunction*:ab,ti OR deficienc*:ab,ti OR impairment*:ab,ti OR locomote*:ab,ti OR physiological:ab,ti OR resilience:ab,ti OR cognitive:ab,ti OR psychological:ab,ti OR psychiatry:ab,ti OR sensory:ab,ti OR social:ab,ti OR integration:ab,ti OR vitality:ab,ti OR fatigue:ab,ti OR positive:ab,ti OR healthy:ab,ti OR successful:ab,ti OR active:ab,ti OR activity:ab,ti | 18,091,786 results |
| Embase (6 mai) : 10,401 résultats -> Si limite ≥ 2022 : 3,172 results | | | | | |
|  |  | | | | |
| CINAHL (EbscoHOST) | | #1 |  | MH "Patient-Reported Outcomes" OR MH "Outcomes (Health Care)" OR MH "Process Assessment (Health Care)" OR MH "Outcome Assessment" OR MH "Artificial Intelligence+" OR MH "Machine Learning+" OR MH "Clinical Prediction Rules" OR XB (scor* OR test* OR assessment* OR evaluation* OR index OR measure* OR PROM OR PROMs OR PRO OR PROs OR patient* N3 outcome* OR rating* OR questionnaire* OR survey* OR scale* OR instrument* OR monitoring OR indices OR indicator* OR "clinical prediction model*" OR "machine learning" OR "artificial intelligence" OR "deep learning" OR "deep-learning") | 2 764 341 |
|  | |  | AND |  |  |
|  | | #2 |  | TI (validat* OR approval* OR approved* OR demonstrat*) | 59 365 |
|  | |  | AND |  |  |
|  | | #3 |  | MH "Geriatric assessment" OR MH "Activities of Daily Living" OR MH "Geriatric Functional Assessment" OR MH "OARS Multidimensional Functional Assessment Questionnaire" OR MH "Aging+" MH "Aged+" OR MH "Geriatrics" OR MH "Longevity" OR MH "Cognitive Aging" OR XB (aging OR ageing OR aged OR elder* OR older OR decline OR senil* OR senior OR "advanced age" OR geriatric*) | 694 517 |
|  | |  | AND |  |  |
|  | | #4 |  | MH "Quality of Life" OR MH "Healthy Aging" OR MH "Activities of Daily Living" OR MH "Healthy Life Expectancy" OR MH "Cognition" OR MH "Physical Mobility" OR XB (intrinsic OR physical OR "well being" OR qualit* N3 life OR QOL OR functional OR detection OR prevention OR frail* OR abilit* OR capacit* OR disabilit*) | 1 383 429 |

# 7 mai 2025 : #1 AND #2 AND #3 AND #4 : 2464 results

# Si limite ≥ 2022 : 559 results

| PsycINFO (ovid) |  | treatment outcomes/ or artificial intelligence/ or machine learning/ or (scor* or test* or assessment* or evaluation* or index or measure* or PROM or PROMs or (patient adj4 outcome*) or rating* or questionnaire* or survey* or scale* or instrument* or monitoring or indices or indicator* or clinical prediction model* or machine learning or artificial intelligence or deep learning).ab,ti. | 2729757 |
| --- | --- | --- | --- |
|  | AND |  |  |
|  |  | (validat* or approv* or demonstrat*).ti. | 40595 |
|  | AND |  |  |
|  |  | geriatric assessment/ or aging/ or cognitive aging/ or geriatric patients/ or geriatric psychiatry/ or geriatrics/ or gerontology/ or (aging or ageing or aged or elder or older or elderly or decline or senile or senility or senior or "advanced age").ab,ti. | 606032 |
|  | AND |  |  |
|  |  | "quality of life"/ or "health related quality of life"/ or healthy aging/ or cognitive ability/ or cognitive aging/ or health attitudes/ or well being/ or "activities of daily living"/ or activity level/ or geriatric assessment/ or physical mobility/ or social functioning/ or diminished capacity/ or (intrinsic or physical or "well being" or "well-being" or "quality adj4 life" or QOL or functional or detection or prevention or frail* or abilit* or capacit* or disabilit* or disorder* or dysfunction* or deficienc* or impairment* or locomotor or locomotion or physiological or resilience or cogniti* or psychologic* or psychiatry or sensory or social or integration or vitality or fatigue or positive or healthy or successful or active or activity).ab,ti. | 3500026 |

PsycInfo [[1806 to April 2025 Week 4](https://ovidsp.dc1.ovid.com/ovid-new-a/ovidweb.cgi?&S=DJAEFPDJFNACIPAIKPHJFHPKOAOOAA00&Database+Field+Guide=8)] (7 mai) : 3481 résultats

≥ 2022 : 665

-------------------------------------
