# Supplementary material for: Existing validated tools for assessing intrinsic capacity and its components within a positive ageing framework: a scoping review and structured appraisal by the Multidisciplinary International Positive Ageing Group
Source: Age Ageing. 2026 Sep 21;55(9):afag274. doi: 10.1093/ageing/afag274 (PMC13591483; doi:10.1093/ageing/afag274)
Supplement: Supplementary file 1 [file supplementary_material_afag274.zip › aa-26-1600-File006.docx]

**Existing validated tools for assessing intrinsic capacity and its components within a positive ageing framework: A scoping review and structured appraisal by the Multidisciplinary International Positive Ageing Group (MIPAG)**

**Appendix 2: Full Method**

**2.1. Study Design**

This scoping review was conducted following the Preferred Reporting Items for Systematic Reviews and Meta-Analyses extension for Scoping Reviews (PRISMA-ScR) [15]. The protocol was registered on the Open Science Framework (OSF: registration ID: https://doi.org/10.17605/OSF.IO/WHTZR).

## 2.2. Eligibility Criteria

To provide comprehensive coverage while avoiding duplication of an existing high-quality review, we adopted an updated scoping review approach. We built upon the scoping review by López-Ortiz et al., which identified studies published before January 2022, and performed an updated English written literature search covering the period from January 2022 to May 2025. The studies included in the previous review that met our eligibility criteria were incorporated into the present analysis, resulting in a comprehensive synthesis of the available evidence. Studies had to assess or measure IC and its domains in adults and report the results of a validation study of one or more tools assessing at least one of the five key areas of IC (locomotor, vitality, sensory, cognition, and psychological). No geographic limitation was applied. Eligible instruments included questionnaires, performance-based tests, composite indices, prediction models, risk scores, and algorithms that were developed or used to assess, estimate, or operationalize IC or one or more of its domains.

Publications were excluded if they did not provide data on validated instruments or outcomes (e.g., non-validated scores or disease-specific scores), or if the tools were used exclusively at the population level (e.g., for public health policy purposes). Books, editorials, letters, official websites, and case reports were also excluded.

**2.3. Information Sources and Search Strategy**

An initial exploratory search was conducted to identify controlled-vocabulary terms and keywords used in the titles and abstracts of relevant literature. A comprehensive literature search strategy was then developed in collaboration with an information specialist (VS) and refined through discussions with the expert consultation MIPAG team. The strategy combined MeSH terms and free-text keywords and was first developed and tested in Medline (PubMed) to ensure accuracy, sensitivity, and comprehensive topic coverage by verifying the retrieval of relevant key articles. The final search strategy focused on concepts related to "healthy ageing" AND ("score" OR "questionnaire"), as detailed in supplementary file 1.

This finalized search strategy was applied to four bibliographic databases on May 7, 2025: Medline (PubMed interface), Embase (Elsevier interface), CINAHL (EBSCOhost platform), and PsycINFO (Ovid). No filters were applied to the search strategy. Additionally, the reference lists of included studies were examined to identify any additional relevant articles.

All identified bibliographic records were imported into Covidence (Veritas Health Innovation, Melbourne, Australia), and duplicates were removed. The screening process was conducted by two independent reviewers (DD and GF), who assessed titles and abstracts against predefined eligibility criteria. Following the initial selection, all relevant articles underwent full-text screening in accordance with predefined inclusion and exclusion criteria. The reviewers independently selected articles to compile the final list, and any conflicts during screening were resolved through discussion with a third expert (YH).

**2.4. Data Charting Process**

Data were extracted by YH using Covidence and further refined through consultation with the MIPAG. A standardized data charting form was developed to ensure consistency in data extraction.

The data charting form included the following items: general information about the study (first author and year of publication), population characteristics (number of participants, sex, age, health status/pathology), inclusion and exclusion criteria, and presence of a control group, IC domains assessed, name of the assessment tool(s), and key findings.

**2.5 Data items**

To ensure comprehensive coverage of IC assessment tools and considering the existence of previously published reviews with a similar scope, the tools identified in the most recent review [8] were also included in the present analysis. This complementary approach was implemented as a quality assurance strategy to enhance the completeness of the mapping and to ensure that relevant instruments were not overlooked when relying solely on database searches.

For each identified tool, a standardized summary was created, including the first author, year, country of publication, population characteristics, assessed domains, comparators (when applicable), mode of administration, key findings from the validation study, and the online link to the corresponding validation publication.

Beyond descriptive mapping, the identified tools underwent a structured expert evaluation to assess their methodological robustness and practical applicability. Each tool was assessed according to five predefined criteria developed by the MIPAG expert group, with equal weighting across criteria: scientific validity (1–5): evidence of reliability, validity, and sensitivity/specificity; clinical relevance (1–5): suitability for individual-level assessment versus community/population-level assessment; usability in clinical practice (1–5): time required, complexity, training needs, and feasibility in real-world settings; patient self-assessment usability (1-5): extent to which the tool can be used independently by patients without assistance from clinicians or caregivers and suitability for patients younger than 60 years (S60; 1-5): appropriateness, effectiveness, and accessibility of the tool for populations under 60 years old.

The expert panel consisted of nine independent researchers and clinicians with complementary expertise in geriatrics (SD, VB), neurology (GF), physical and rehabilitation medicine (DD), physiotherapy (YH), psychiatry (LA), psychometrics (GB), and the assessment of intrinsic capacity (TC, AJC). To distribute the workload while ensuring independent evaluation, each assessment tool was assigned to two experts according to their area of expertise. Thus, although the panel comprised nine experts overall, each individual tool was evaluated by two independent reviewers. Before the assessment, all experts received a standardized evaluation grid describing the predefined evaluation criteria and scoring instructions to ensure consistency across reviewers. Experts completed their assessments independently using this evaluation grid and were blinded to each other's scores during the initial evaluation. Discrepancies between reviewers were subsequently discussed until consensus was reached. When consensus could not be achieved, a third expert from the panel (YH) independently reviewed the tool and contributed to the final consensus decision. Each criterion was rated on a five-point Likert scale ranging from 1 (weak) to 5 (excellent). Mean scores and standard deviations were calculated for each criterion and for the overall score, with a maximum possible total score of 25.
